# Molecular Dynamics Simulations of 4 *GALC* Variants Causing Krabbe Disease

**DOI:** 10.34133/csbj.0101

**Published:** 2026-05-20

**Authors:** Piet Ankermann, Silja I. Jenne, Jannes Talarek, Lukas Heger, Eileen Socher

**Affiliations:** ^1^ Institute of Functional and Clinical Anatomy, Friedrich-Alexander-Universität Erlangen-Nürnberg (FAU), Erlangen, Germany.; ^2^ Department of Transfusion Medicine and Hemostaseology, Universitätsklinikum Erlangen, Friedrich-Alexander-Universität Erlangen-Nürnberg (FAU), Erlangen, Germany.

## Abstract

Krabbe disease, also known as globoid cell leukodystrophy, is a rare lysosomal disorder affecting the white matter of the central and peripheral nervous systems. It is characterized by neurodegeneration, with infantile-onset Krabbe disease as the most common form, typically diagnosed within the first year of life and associated with high morbidity and mortality. This autosomal recessive disease is caused by mutations in the *GALC* gene, which encodes the lysosomal enzyme β-galactocerebrosidase (GALC). This study focuses on examining the structural effects of 4 GALC variants (Gly59Arg, Ser68Phe, Thr278Ile, and Ser303Phe) found as mutations in the *GALC* gene of patients with Krabbe disease. To investigate the effects of these mutations on protein structure, structural models of human GALC were built. These models served as the basis for a series of all-atom molecular dynamics (MD) simulations to analyze the structural stability of the wild type and the mutated enzyme variants. Since GALC is subcellularly localized in the lysosome (pH 4.5 to 5.5), MD simulations were performed with protonation states corresponding to pH 4.5. Differences in protein flexibility and intramolecular interactions between the wild type and the variants were observed. Similarly, we detected the effects of the mutations on the substrate-binding residues and their vicinity, although the mutation sites themselves do not lie within the active site/binding site of the enzyme. Overall, our MD simulations shed light on how these mutations affect the structure of human GALC in the lysosome and offer possible explanations as to why these mutations have an effect on enzyme function.

## Highlights

• We performed molecular dynamics simulations for 4 missense mutations in β-galactocerebrosidase (GALC) causing Krabbe disease.
• The Gly59Arg variant of GALC demonstrates enhanced hydrogen bonding and the presence of an additional cation–π interaction.
• Ser68Phe mutation increases the size of the substrate-binding pocket in GALC.
• All 4 mutations affect the β-hairpin structure surrounding substrate-binding Thr109.
• Ser303Phe mutation destabilizes the β-sheet formation of the triosephosphate isomerase barrel.

## Graphical Abstract

**Figure:**
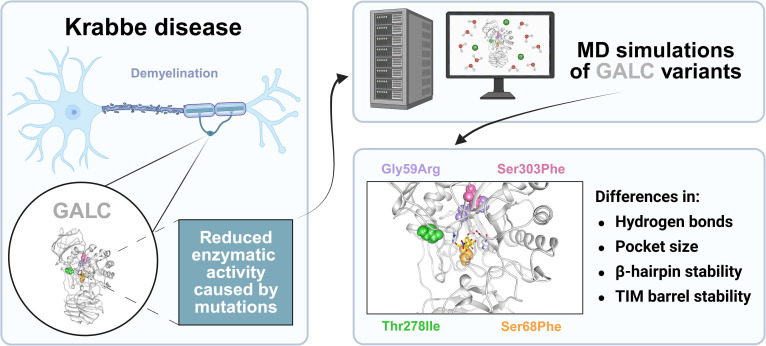

## Introduction

Lysosomal storage diseases are a group of over 70 diseases characterized by defects in lysosomal function. Although these disorders are individually rare, their combined prevalence is estimated to be 1 in 5,000 live births [1]. Most lysosomal storage diseases are inherited in an autosomal recessive manner. This is likewise the case for the devastating neurodegenerative Krabbe disease (KD), also known as globoid cell leukodystrophy or galactosylceramide lipidosis, which belongs to the lysosomal storage disease subclass of sphingolipidoses.

KD is caused by the deficiency of the lysosomal enzyme β-galactocerebrosidase (GALC, also known as galactosylceramidase) due to mutations in the *GALC* gene. The enzyme GALC degrades complex galactosides, carbohydrate molecules such as galactocerebrosides and galactosylsphingosine, to provide metabolites, such as sphingosine and ceramide. These are critical for the synthesis and maintenance of myelin, the insulating sheath that improves electrical transmission along neurons. The functional deficiency of GALC leads to the accumulation of its substrates, particularly the toxic galactosylsphingosine (psychosine), causing degeneration of oligodendrocytes and thus severe demyelination [2,3].

Current therapeutic strategies for KD, such as hematopoietic stem cell transplantation, primarily focus on delaying disease progression and mitigating clinical symptoms [4]. Experimental approaches, including gene therapy, enzyme replacement therapy, and substrate reduction therapy [5–7], have shown potential in animal models but yet lack long-term clinical validation in human patients [8–10]. Thus, there remains a critical need for innovative therapeutic modalities, with the development of small-molecule activators that enhance residual GALC enzyme activity. Such compounds could stabilize the enzyme’s 3-dimensional structure or promote proper folding, thereby improving its function in patients with partially active variants as shown for the structurally related lysosomal enzyme glucocerebrosidase [11,12]. For the rational design of similar activators targeting GALC, it is crucial to obtain detailed structural insights into the enzyme itself and to understand how pathogenic mutations alter its conformation and dynamics. Structural biology, computational modeling, and pharmacological screening therefore represent essential tools to identify and optimize candidate molecules that could augment GALC activity and complement existing therapeutic strategies such as gene or stem cell therapy.

In a recently published study, we showed by using structural modeling and molecular dynamics (MD) simulations that the pathogenic missense variant Thr112Ala [13,14] of GALC significantly changes the conformational stability of a β-hairpin around the substrate-binding residue Thr109 and that the mutation also changes the volume of the substrate-binding pocket [15]. Hence, we could provide a possible structural explanation of how the GALC variant Thr112Ala influences the enzymatic activity [15].

As a multitude of GALC variants with unknown pathogenicity exist, the aim of the study presented here was to provide structure-based explanations of how mutations in the vicinity of the active center of GALC, although not directly affecting it, can lead to KD. We investigated the 4 pathogenic missense variants Gly59Arg [16], Ser68Phe [16], Thr278Ile [16], and Ser303Phe [17,18] by using structural modeling and MD simulations. The variants Gly59Arg, Ser68Phe, and Thr278Ile were originally identified in heterozygous patients with infantile-onset KD [16], and *in vitro* overexpression experiments demonstrated a complete loss of enzyme activity, consistent with a severe infantile phenotype. The Ser303Phe variant has been reported in both a homozygous Saudi Arabian patient and in compound-heterozygous individuals with infantile KD [17,18], indicating that this mutation similarly results in the loss of GALC activity. Notably, none of these 4 mutations directly affect residues involved in substrate binding or catalysis. This suggests that their pathogenicity, like the previously investigated Thr112Ala variant, arises from indirect structural effects, such as changes in conformational stability, altered interdomain hydrogen bonds, or impaired folding and dynamics of the GALC protein. Understanding the specific effects of these missense mutations on GALC enzyme structure and function is important for developing targeted therapies, such as enzyme activators, and underscores the need for personalized approaches based on the mutation and enzyme activity profile. By performing structural modeling and MD simulations, we provide structure-based explanations of how the individual GALC variants might reduce enzymatic activity of GALC.

## Materials and Methods

### Generation of the starting structures

The starting structures for the MD simulations of wild-type GALC and all 4 GALC variants were generated with AlphaFold [19]. Subsequently, the reliability of these *in silico* generated structures was evaluated by superimposing them with the experimentally resolved x-ray crystal structure of the murine GALC (Protein Data Bank [PDB] ID: 3ZR5 [20]; root-mean-square deviation [RMSD]: below 0.9 Å; 83% sequence identity with human GALC). The starting structure of GALC was prepared without a bound ligand in order to observe the substrate-binding pocket in its full range of structural flexibility. This means that by studying GALC without a ligand attached, we aimed to capture the enzyme’s binding pocket in all of its possible conformations, not limited or influenced by ligand binding. In contrast, structures solved or simulated with ligands usually show the pocket in a (single) ligand-bound conformation.

Since GALC is a lysosomal enzyme (pH 4.5 to 5.5) and the pH profile of enzyme activity measured for GALC showed the maximum activity close to pH 4.5 [21], protonation states corresponding to pH 4.5 were defined by using the “PQR” output files generated by the APBS-PDB2PQR software suite [22] (https://server.poissonboltzmann.org/pdb2pqr) and subsequent verification by manual inspection, taking the local environment into account. The aspartate side chain involved in the coordination of the calcium ion (Ca^2+^) was always retained in its deprotonated carboxylate state.

### All-atom MD simulations

The MD simulations were conducted very similarly to previously described protocols [15,23–26], using version 22 [27] of the Amber Molecular Dynamics software package (http://ambermd.org) and the ff14SB force field [28]. During the preparation with the Amber tool LEaP, all systems were electrically neutralized with chloride (Cl^−^) ions and solvated using TIP3P [29] water molecules. The GALC enzyme was solvated in a water box with the shape of a truncated octahedron and a minimum distance of 25 Å between the solute and the box boundaries.

The MD simulations were executed following a previously established and validated protocol. Energy minimization was performed in 3 consecutive stages to optimize the geometry of the initial structures. In the first stage, only water molecules were minimized, while all other atoms were restrained to their initial positions using a harmonic force constant of 10 kcal mol^−1^ Å^−2^. During the second stage, chloride (Cl^−^) ions and the protein’s hydrogen atoms were allowed to relax, whereas the remaining protein atoms were kept restrained with the same force constant. In the final minimization stage, all restraints were released, allowing the protein, ions, and solvent molecules to relax freely. Each minimization stage consisted of 2,500 steps using the steepest descent algorithm followed by 2,500 steps employing the conjugate gradient method. The equilibrations were carried out in 2 phases. First, the temperature was gradually increased from 10 to 310 K over 0.1 ns while maintaining positional restraints on all protein atoms (5 kcal mol^−1^ Å^−2^). In the second phase, which lasted 0.4 ns, only the Cα atoms were restrained with the same force constant. Both equilibration steps were conducted using a 2-fs integration time step. Minimization and equilibration were performed on central processing units, while production runs were executed with pmemd.CUDA on Nvidia A40 graphics processing units [30–32]. The production simulations, each lasting 1,000 ns, were conducted without restraints at 310 K using a Berendsen thermostat [33]. Constant-pressure periodic boundary conditions (1 bar, isotropic scaling) were applied throughout. The SHAKE algorithm [34] was employed to constrain bonds involving hydrogen atoms during the equilibration and production phases. For statistical robustness, 4 independent 1,000-ns-long production runs were performed for the wild-type GALC and for each of the 4 GALC variants.

Trajectory analysis (analysis of electrostatic linear interaction energies; analysis of RMSD for the backbone atoms N, Cα, and C; analysis of root-mean-square fluctuations [RMSFs; by residue, restricted to the backbone atoms N, Cα, and C], analysis of contacts [always with a distance criterion of ≤5 Å between any pair of atoms; total fraction of contacts for residue pairs], measurement of interatomic distances, analysis of hydrogen bonds, and analysis of the secondary-structure elements [average secondary structural propensities over all frames for each residue; using the Define Secondary Structure of Proteins (DSSP) method of Kabsch and Sander]) was performed using the Amber tool cpptraj [35].

As reference for assuming a cation–π interaction between the side chains of Arg59 and Trp271, a distance of 3.7 Å between the NH1, NH2, and CZ atoms of the arginine side chain and the benzene ring of the tryptophan side chain was used [36].

### Calculating the pocket volume with POVME

Pocket volume analyses were performed using POVME (Pocket Volume MEasurer) 2.0, a grid-based method for the quantitative characterization of binding-site volumes [37]. The MD trajectories of the wild-type protein and the corresponding variants were used as input. From each trajectory, every hundredth frame was extracted, yielding 251 individual PDB structures per system. The pocket was defined based on the residues building the active site and substrate-binding pocket (Gly64, Thr109, Trp151, Asn197, Glu198, Glu274, Ser277, Trp307, Tyr319, Arg396, Trp540, and Ile586) [38], with the pocket center positioned at the center of mass of these residues, calculated in the first trajectory frame using UCSF Chimera 1.19 [39]. An inclusion sphere was defined based on this center, applying a radius of 9 Å. Identical pocket definitions and POVME parameters were applied to both the wild-type and mutant systems to ensure direct comparability. Pocket volumes were calculated for each extracted structure using POVME 2.0, and the resulting volume distributions were subsequently compared between the 2 systems.

### Statistics and display

Statistical analyses were performed with GraphPad Prism (version 10.6.1 for Windows; GraphPad Software, www.graphpad.com), and statistical tests were applied as indicated below the figures. Plots were created in GraphPad.

Images of protein structures were made with UCSF Chimera 1.19 [39] or UCSF ChimeraX 1.10.1 [40–42].

The sequence alignment annotated with secondary-structure elements (Fig. S1) was visualized using ESPript 3.2 (https://espript.ibcp.fr/ESPript/ESPript; accessed 2026 January 20) [43], based on the sequence alignment of murine GALC (UniProtKB entry: P54818) and human GALC (UniProtKB entry: P54803) and the experimentally determined murine GALC protein structure (PDB ID: 4CCC [21]).

Conservation plots were created with BioEdit (version 7.2.5) by using multiple sequence alignments generated with the Clustal Omega program (version 1.2.4) [44].

The following sequences were used as homologs for human GALC (UniProtKB entry: P54803): *Pan troglodytes* (UniProtKB entry: H2Q8Q9), *Pongo abelii* (UniProtKB entry: A0A2J8W5I2), *Callithrix jacchus* (UniProtKB entry: F7HLB5), *Otolemur garnettii* (UniProtKB entry: H0WHI3), *Mus musculus* (UniProtKB entry: P54818), *Rattus norvegicus* (UniProtKB entry: A6JEE0), *Oryctolagus cuniculus* (UniProtKB entry: G1SWZ5), *Canis lupus familiaris* (UniProtKB entry: P54804), *Felis catus* (UniProtKB entry: A0A337SQ01/UniParc UPI000C2FAC05), *Capra hircus* (UniProtKB entry: A0A452F6F4), *Bos taurus* (UniProtKB entry: A0AAA9SDL9), *Sus scrofa* (UniProtKB entry: F1SDZ2), *Balaenoptera musculus* (UniProtKB entry: A0A8C0HYA9), *Equus caballus* (UniProtKB entry: A0A9L0RMH0), *Myotis lucifugus* (UniProtKB entry: G1PV17), *Loxodonta africana* (UniProtKB entry: G3SWK6), *Vombatus ursinus* (UniProtKB entry: A0A4X2LCI5), *Sarcophilus laniarius* (UniProtKB entry: A0A7N4NKH4), *Ornithorhynchus anatinus* (UniProtKB entry: F6SXG1), and *Danio rerio* Galca (UniProtKB entry: Q5SNX7) and Galcb (UniProtKB entry: Q7ZUD5).

## Results

**Fig. 1.**
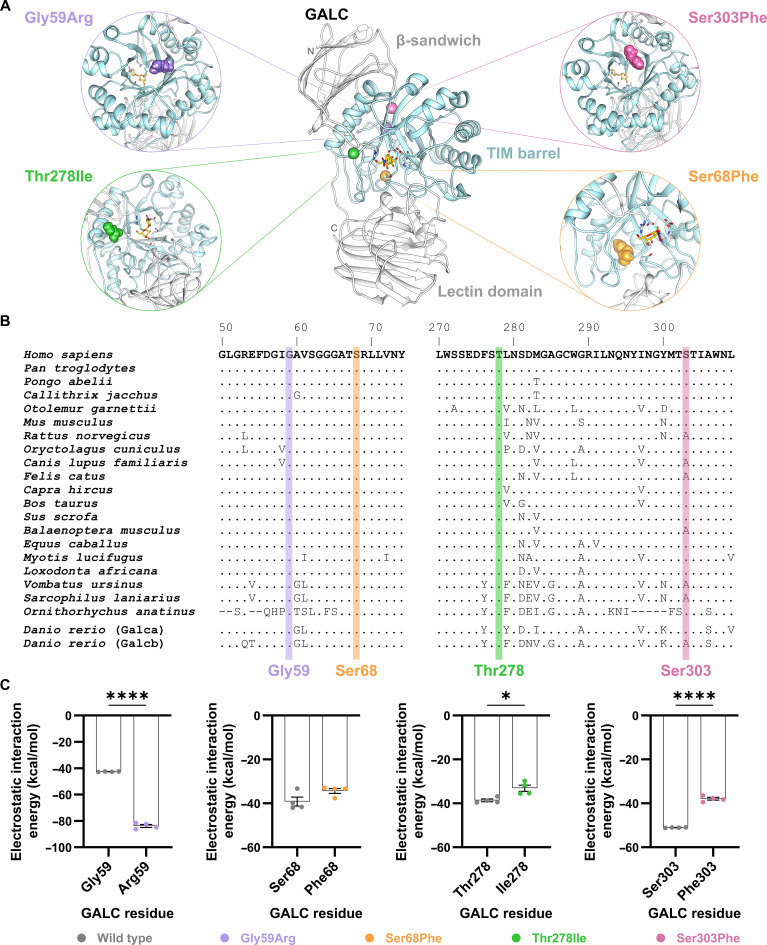
Structural representation of the β-galactocerebrosidase (GALC) protein, conservation, and electrostatic linear interaction energies of the mutation sites. (A) Overview of the human GALC structure showing the 4 investigated mutations (Gly59Arg, Ser68Phe, Thr278Ile, and Ser303Phe), which are all in the central triosephosphate isomerase (TIM) barrel (light blue) as well as the catalytic or substrate-binding residues and a substrate molecule (yellow, shown as sticks), which was inserted in order to highlight the position of the GALC active site. Mutated residues are displayed with spheres: Arg59 (purple), Phe68 (orange), Ile278 (green), and Phe303 (pink). The structures in the enlargements were rotated to better visualize the mutation sites and their structural vicinity. (B) Conservation plots show the amino acid sequence segments surrounding the mutation sites in human GALC as well as the conservation in respective homologs. Amino acids are shown in the one-letter code, and periods indicate evolutionarily conserved residues in the respective homologs of human GALC. Amino acid exchanges are represented by the one-letter code. Residue numbers of the human GALC are depicted on top of the human sequence regions. (C) Electrostatic linear interaction energies of the mutated residue in the GALC variants (colored) and the corresponding residue in wild-type GALC (gray). Each circle represents an independent simulation run (*n* = 4 for wild type and each variant). Statistical analysis was performed in GraphPad Prism (V10) using an unpaired *t* test (*P < 0.05; ****P < 0.0001).

GALC is a multidomain lysosomal enzyme composed of a β-sandwich domain, a central triosephosphate isomerase (TIM) barrel, and a C-terminal lectin domain (Fig. 1A). The β-sandwich domain forms a structural scaffold and contributes a critical loop region to the substrate-binding pocket, including an arginine residue stabilizing galactose binding by hydrogen bonding. The central TIM barrel constitutes the catalytic core of GALC, containing the conserved glutamate residues Glu198 and Glu274 that mediate glycosidic bond hydrolysis via a retaining mechanism. The C-terminal lectin domain represents a previously uncharacterized feature among related hydrolases and is proposed to participate in carbohydrate recognition, enzyme trafficking, and stabilization of the overall domain architecture [20].

For this study, we selected 4 pathogenic missense variants of GALC: Gly59Arg, Ser68Phe, Thr278Ile, and Ser303Phe. All of these variants are associated with a severe loss of enzyme activity and therefore lead to the infantile form of KD [16–18]. Moreover, all 4 mutation sites are located in the central TIM barrel (residues 57 to 353) [20] of GALC and they are in structural proximity to the catalytic and substrate-binding residues. However, the mutated residues do not directly contribute to the binding of the substrate (Fig. 1A and Fig. S1). All 4 mutation sites are highly conserved in mammals and even in the zebra fish Galca (UniProtKB entry: Q5SNX7) and Galcb proteins (UniProtKB entry: Q7ZUD5) (Fig. 1B). Furthermore, the selected mutations have in common that they change the biophysical properties of the side chain at the mutation site either from a tiny uncharged side chain to a large charged side chain (Gly59Arg) or from a polar uncharged side chain to a hydrophobic side chain (Ser68Phe, Thr278Ile, and Ser303Phe). We confirmed this change of the biophysical side-chain properties by determining the linear electrostatic interaction energies (Fig. 1C). The missense mutation Gly59Arg led to significantly more negative electrostatic linear interaction energies with regard to the interaction between the amino acid at position 59 and the remaining protein due to the large, charged arginine side chain. The loss of the polar side chain in all other investigated GALC variants (Ser68Phe, Thr278Ile, and Ser303Phe) resulted in higher electrostatic interaction energies (less favorable values). However, the change in electrostatic interaction energies was not significant for Ser68Phe (Fig. 1C). Due to the nature of these substitutions, all 4 mutations probably would lead to a cascade of structural changes. These include alterations in the hydrogen-bond network within the protein and specific secondary-structure motifs compared to the wild type, affecting both residues within the substrate-binding pocket and those flanking the modified region. These findings might provide insights into how each of the variants leads to the development of KD.

### Global and local structural impact of GALC mutations

To understand how individual mutations affect the protein structure of GALC, we analyzed both global and local structural changes. At the global level, we examined alterations in the overall protein stability (backbone fluctuations of the entire protein). At the local level, we focused on structural changes in the immediate vicinity of each mutation site. In particular, we assessed how the mutated residue reshaped its network of contacts with neighboring residues, for example, through side-chain-mediated interactions such as hydrogen bonds or cation–π interactions.

#### Global effect of the mutations: Different protein flexibility

**Fig. 2.**
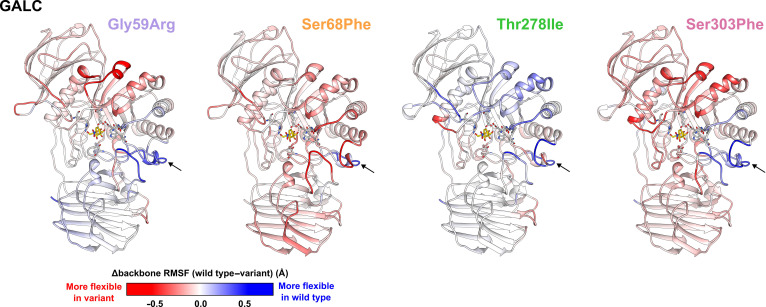
Comparison of residue backbone fluctuations between wild type and β-galactocerebrosidase (GALC) variants. Representation of GALC colored according to changes in residue backbone fluctuations (Δbackbone root-mean-square fluctuation [RMSF]). Each structure corresponds to 1 of the 4 investigated mutations. Coloring reflects the difference in RMSF of the backbone atoms of each residue between the GALC variant and the wild-type GALC, calculated as mean of the 4 simulation runs. Red indicates increased backbone fluctuations (negative RMSF difference), whereas blue indicates decreased backbone fluctuations (positive RMSF difference) compared to the wild-type protein. The color scale is capped at ±0.5 Å, such that any residue with an RMSF difference of ≤−0.5 or ≥0.5 Å is colored red or blue, respectively. All 4 GALC variants showed a decreased backbone fluctuation for the loop region harboring residues Ser115 to Asn124 (denoted with an arrow). To facilitate visual orientation in the structure, the position of the GALC active site was highlighted by the insertion of a substrate molecule.

First, we determined the RMSD values for all protein backbone atoms for the wild type and each GALC variant over the complete simulation time (Figs. S2 and S3). Each individual simulation run showed stabilization with maximal RMSD values below 4 Å (Figs. S2 and S3). All 4 investigated variants exhibited an overall comparable backbone flexibility, with localized regions showing either increased or decreased fluctuations (Fig. 2). Interestingly, these changes were not restricted to areas near the mutation sites but were distributed throughout the entire protein structure, suggesting a global impact on protein dynamics. The backbone flexibility of the β-sandwich domain was only slightly increased in the GALC variants (Gly59Arg, Ser68Phe, and Ser303Phe) or it was not altered (Thr278Ile). In the central TIM barrel, all 4 GALC variants showed a decreased backbone fluctuation for the loop region harboring residues Ser115 to Asn124 (Fig. S4). In the C-terminal lectin domain, backbone fluctuations were slightly increased for Ser68Phe and Ser303Phe, unchanged for Thr278Ile, and slightly decreased for Gly59Arg. Additional minor local changes in backbone flexibility compared to the wild type were observed throughout the protein (Fig. 2). As the mutations might have stronger effects on residues in their vicinity, we next analyzed local effects for each individual variant.

#### Local effects of the GALC variant Gly59Arg

**Fig. 3.**
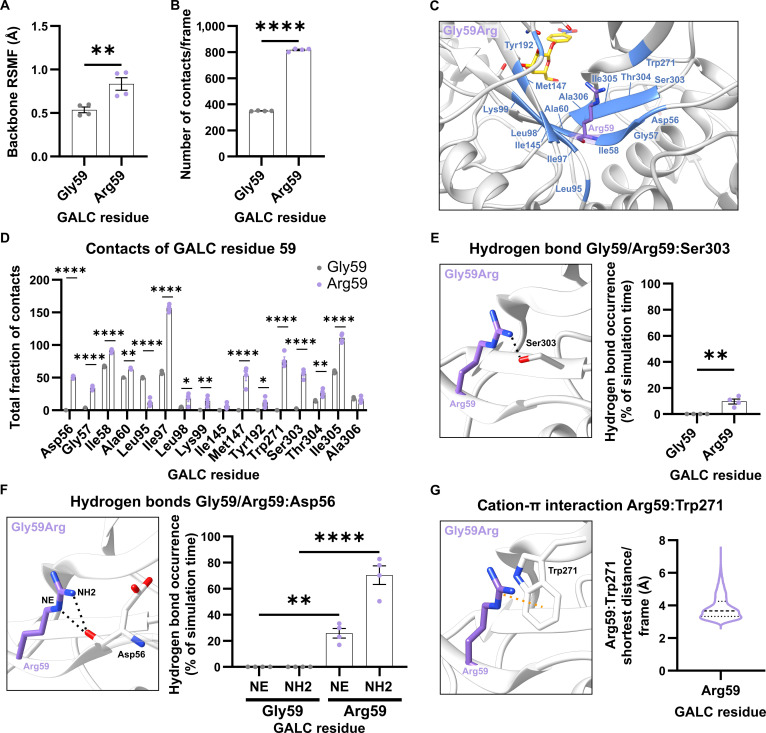
Local effects of the mutation in the β-galactocerebrosidase (GALC) variant with Gly59Arg. (A) Root-mean-square fluctuation (RMSF) of the backbone atoms of Gly59 (wild-type GALC) and Arg59 (GALC variant Gly59Arg). (B) Number of contacts formed by Gly59/Arg59 with other residues in GALC. (C) Structural representation of residues in close contact (colored blue) with Gly59 or Arg59. To facilitate visual orientation in the structure, the position of the GALC active site was highlighted by the insertion of a substrate molecule. (D) Residue-specific analysis of the total fraction of contacts between Gly59 or Arg59 and their interaction partners within GALC. (E) Structural representation of the hydrogen bond (indicated by a black dashed line) between the hydroxyl group of Ser303 and the guanidino group of Arg59 (black dashed line). This interaction is absent in wild-type GALC due to the lack of a guanidino group in Gly59. Hydrogen-bond occurrence was measured for each simulation run of the wild type and the GALC variant with Gly59Arg. (F) Structural representation of the hydrogen bonds (indicated by black dashed lines) between the Arg59 side chain and the backbone of Asp56. This interaction is absent in wild-type GALC for the same reason as above. Hydrogen-bond occurrence was measured as in (E). (G) Structural representation of the cation–π interaction between the indole ring of Trp271 and the positively charged guanidinium group of Arg59 (orange dashed line). In addition, a violin plot (dashed line: median; dotted lines: quartiles) is shown displaying all data points from the 4 GALC variant Gly59Arg simulation runs, illustrating the shortest distance of the 3 distances between the benzene ring of the tryptophan side chain and the NH1, NH2, and CZ atoms of the arginine side chain. These distances were measured for each frame, and the minimum distance per frame was plotted. This interaction is absent in the wild-type GALC protein. (A, B, and D to F) Mean values are shown as bar graphs ± standard error of the mean (SEM) with each circle representing an individual simulation run (*n* = 4). Statistical testing was performed in GraphPad Prism (V10) using (A, B, and E) an unpaired *t* test, (D) 2-way analysis of variance (ANOVA) with Šídák’s multiple comparisons test, or (F) one-way ANOVA with Šídák’s multiple comparisons test or (*P < 0.05; **P < 0.01; ****P < 0.0001).

The Gly59Arg mutation is located within one of the parallel β-strands of the TIM-barrel structure (Fig. 1A). The substitution of glycine with the bulkier, positively charged arginine at position 59 resulted in significant elevated backbone fluctuations (Fig. 3A) and increased local contacts (distance criterion: 5 Å; Fig. 3B). To identify the interaction partners of the residue at position 59, we analyzed with which other amino acids from GALC the Gly59 in the wild type or the Arg59 in the GALC variant interacts (Fig. 3C and D). Only the most prominent interactions in at least one of the simulation runs were included in this analysis. This analysis revealed a higher total fraction of contacts for the residues immediately adjacent to the mutation site (Asp56, Gly57, Ile58, and Ala60) or in the 2 neighboring β-strands of the inner β-barrel of the TIM barrel (Ile97, Leu98, Lys99, Ser303, Thr304, and Ile305), as well as significantly more contacts with Trp271 (Fig. 3D). By analyzing the structure-stabilizing interactions formed by the residues at position 59, we found that the Gly59Arg variant altered the hydrogen-bonding pattern by slightly increasing the likelihood of forming a hydrogen bond with Thr304 (Fig. S5a) and by inducing new hydrogen bonds between the arginine side chain and the side chains of Ser303 (Fig. 3E and Fig. S5b) or the backbone of Asp56 (Fig. 3F and Fig. S5c), which cannot be formed with a glycine at this position in the wild type. Furthermore, the arginine side chain in the GALC variant with Gly59Arg also forms a structure-stabilizing cation–π interaction between the cationic guanidinium group of the side chain of Arg59 and the π system of the nearby side chain of Trp271 (Fig. 3G and Fig. S5d and e). The median of the shortest distance per frame of the 3 distances between the benzene ring of the tryptophan side chain and the NH1, NH2, and CZ atoms of the arginine side chain was 3.66 Å. This allows the assumption that the cation–π interaction persisted for more than half of the simulation time [36], as exemplarily shown in the distance plot of one simulation run (Fig. S5e). Overall, the Gly59Arg variant showed strongly increased interaction with residues in the vicinity that might affect the structure and flexibility of the TIM barrel and the binding pocket.

#### Local effects of the GALC variant Ser68Phe

**Fig. 4.**
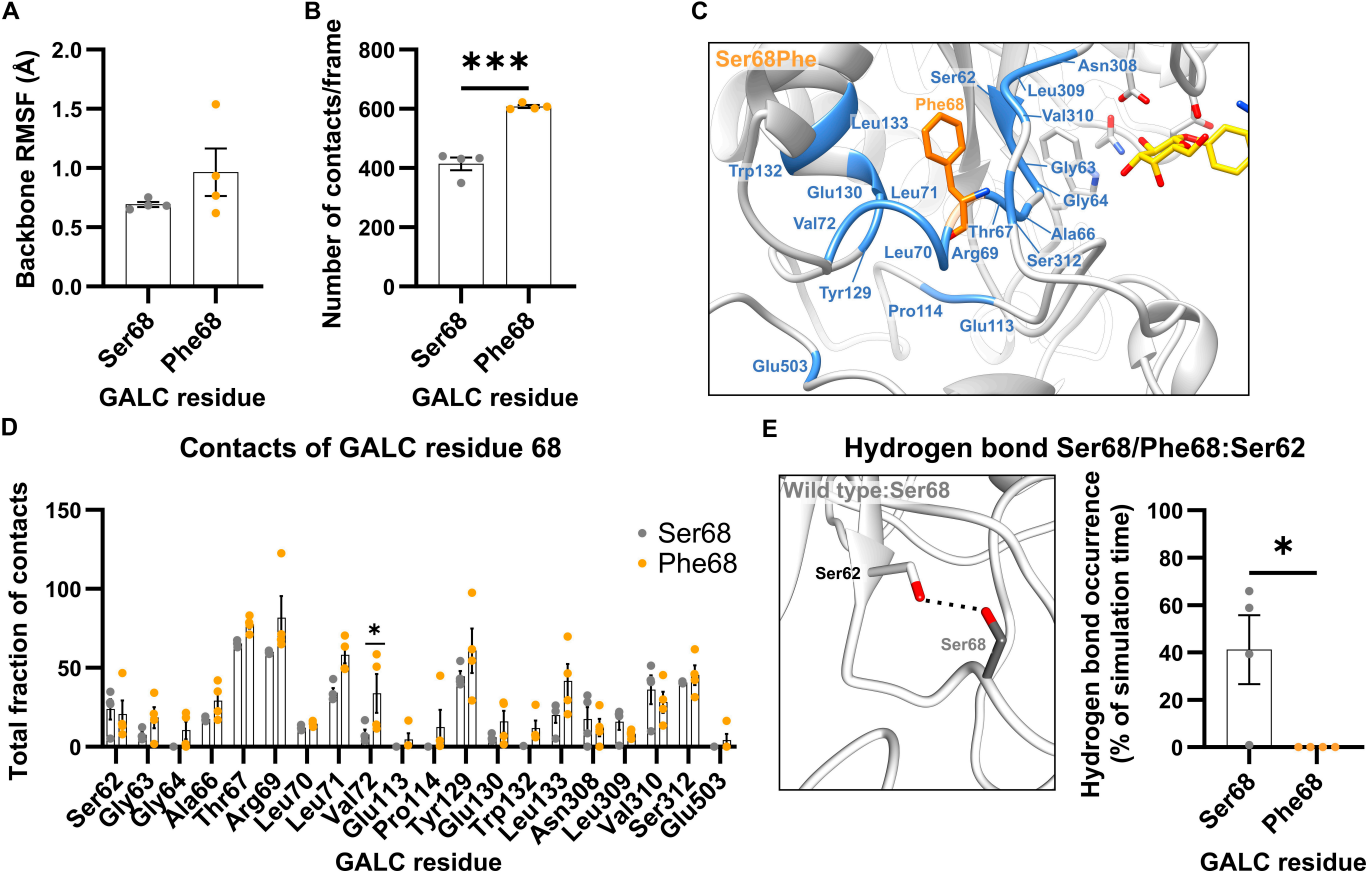
Local effects of the mutation in the β-galactocerebrosidase (GALC) variant with Ser68Phe. (A) Root-mean-square fluctuation (RMSF) of the backbone atoms of Ser68 (wild-type GALC) and Phe68 (GALC variant Ser68Phe). (B) Number of contacts formed by Ser68/Phe68 with other residues in GALC. (C) Structural representation of residues in close contact (colored blue) with Ser68 or Phe68. To facilitate visual orientation in the structure, the position of the GALC active site was highlighted by the insertion of a substrate molecule. (D) Residue-specific analysis of the total fraction of contacts between Ser68 or Phe68 and their interaction partners within GALC. (E) Structural representation of the hydrogen bond (indicated by a black dashed line) between the hydroxyl group of Ser62 and the hydroxyl group of Ser68. This interaction is absent in the GALC Ser68Phe variant, as phenylalanine lacks a functional group in the side chain capable of hydrogen-bond formation (see Fig. S6a). Hydrogen-bond occurrence was measured for each simulation run of the wild type and the GALC variant with Ser68Phe. Mean values are shown as bar graphs ± standard error of the mean (SEM) with circles representing individual simulation runs (*n* = 4). Statistical testing was performed in GraphPad Prism (V10) using (A, B, and E) an unpaired *t* test or (D) 2-way analysis of variance (ANOVA) with Šídák’s multiple comparisons test (*P < 0.05; ***P < 0.001).

Unlike the Gly59Arg mutation, the Ser68Phe mutation is not located within 1 of the 8 parallel β-strands of the TIM barrel but in a connecting loop between a β-strand and the following α-helix (Fig. 1A). Compared to the wild type, the Ser68Phe variant exhibited slightly increased backbone fluctuations at the mutation site at position 68 (Fig. 4A), as well as a significantly increased number of contacts per frame to the atoms in structural vicinity (distance criterion: 5 Å; Fig. 4B). The detailed contact analysis revealed that the total fraction of contacts was increased for the most interaction partners of the Phe68 in the mutated variant compared with the residue Ser68 in the wild-type GALC (Fig. 4C and D); however, it was significantly increased only for Val72. For Ser62, Asn308, Leu309, and Val310, a decrease in the total fraction of contacts was observed (Fig. 4D). This decrease in the total fraction of contacts for Ser62 and Asn308 correlates with the loss of side-chain hydrogen bonds through the mutation Ser68Phe. In the wild-type GALC, Ser68 forms side-chain hydrogen bonds with Ser62 (Fig. 4E and Fig. S6a) and Asn308 (Fig. S6b). Since the phenylalanine side chain cannot participate in hydrogen bonding, the Ser68Phe substitution results in the loss of both hydrogen bonds (Fig. S6a and b). In the wild-type GALC, Ser68 also forms hydrogen bonds, by both the backbone and side chain of Ser68, with the side chain of Tyr129. The side chain–side chain hydrogen bond cannot be formed by a phenylalanine in the GALC variant Ser68Phe, and the hydrogen bond between the Ser68/Phe68 backbone and Tyr129 is observed less frequently (Fig. S6c). Overall, the loss of stabilizing side-chain interactions might explain the observed increase in protein flexibility in the Ser68Phe variant (Fig. 2).

#### Local effects of the GALC variant Thr278Ile

**Fig. 5.**
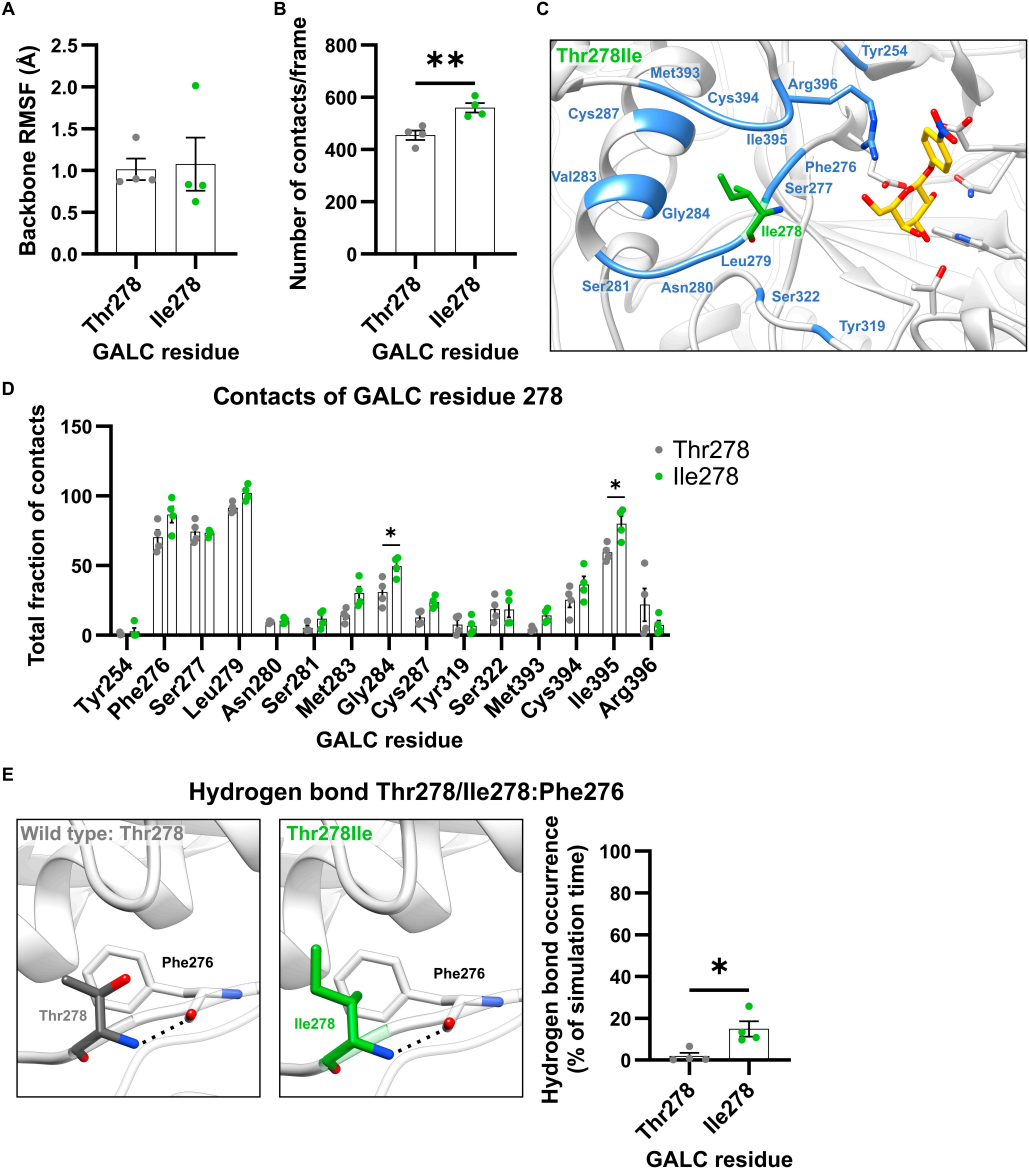
Local effects of the mutation in the β-galactocerebrosidase (GALC) variant with Thr278Ile. (A) Root-mean-square fluctuation (RMSF) of the backbone atoms of Thr278 (wild-type GALC) and Ile278 (GALC variant Thr278Ile). (B) Number of contacts formed by Thr278/Ile278 with other residues in GALC. (C) Structural representation of residues in close contact (colored blue) with Thr278 or Ile278. To facilitate visual orientation in the structure, the position of the GALC active site was highlighted by the insertion of a substrate molecule. (D) Residue-specific analysis of the total fraction of contacts between Thr278 or Ile278 and their interaction partners within GALC. (E) Structural representation of the hydrogen bond (indicated by a black dashed line) between the amino group of Thr278/Ile278 and the backbone carbonyl group of Phe276. Hydrogen-bond occurrence was measured for each simulation run of the wild type and the GALC variant Thr278Ile. Mean values are shown as bar graphs ± standard error of the mean (SEM) with circles representing individual simulation runs (*n* = 4). Statistical testing was performed in GraphPad Prism (V10) using (A, B, and E) an unpaired *t* test or (D) 2-way analysis of variance (ANOVA) with Šídák’s multiple comparisons test (*P < 0.05; **P < 0.01).

Similar to the Ser68Phe mutation, the Thr278Ile mutation is located in a connecting loop between a β-strand and the following α-helix in the TIM barrel (Fig. 1A). While the backbone RMSF value was not different between Thr278 in wild-type GALC and Ile278 in the GALC variant (Fig. 5A), the mean of the number of contacts was significantly higher for Ile278 in the GALC variant Thr278Ile (Fig. 5B). The detailed analysis of the total fraction of contacts of the residue at position 278 revealed that Ile278 has a higher fraction of contacts with Phe276, Leu279, Met283, Gly284, Cys394, and Ile395 and a decreased fraction of contacts with Arg396, which is one of the substrate-binding residues (Fig. 5C and D). The highest values could be measured in wild type and the GALC Thr278Ile variant for the direct neighbors Phe276, Ser277 and Leu279 and for Ile395. With 3 of these residues, the residue Thr278 in wild-type GALC or Ile278 in the GALC variant formed hydrogen bonds. In wild-type GALC, Thr278 formed hydrogen bonds with Leu279 (Fig. S7a) and Ile395 (Fig. S7b) via its side chain. Both hydrogen bonds cannot be formed with an isoleucine at position 278 in the GALC Thr278Ile variant (Fig. S7a and b). In contrast to the hydrogen bonds formed by the side chain of Thr278, the backbone–backbone hydrogen bond between Thr278 and Phe276 could be observed significantly more often, when there was an isoleucine at position 278 (Fig. 5E).

#### Local effects of the GALC variant Ser303Phe

**Fig. 6.**
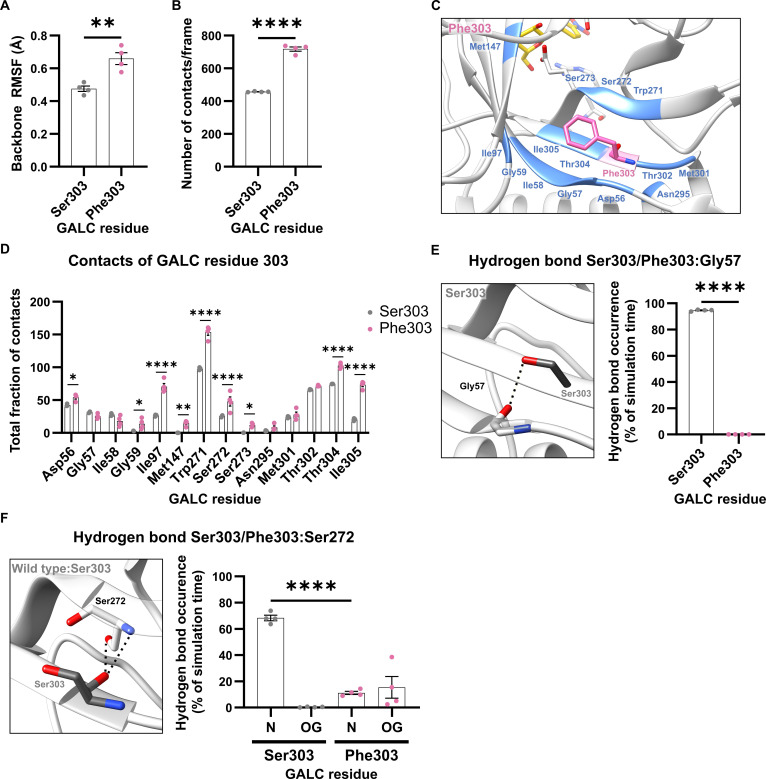
Local effects of the mutation in the β-galactocerebrosidase (GALC) variant with Ser303Phe. (A) Root-mean-square fluctuation (RMSF) of the backbone atoms of Ser303 (wild-type GALC) and Phe303 (GALC variant Ser303Phe). (B) Number of contacts formed by Ser303/Phe303 with other residues in GALC. (C) Structural representation of residues in close contact (colored blue) with Ser303 or Phe303. To facilitate visual orientation in the structure, the position of the GALC active site was highlighted by the insertion of a substrate molecule. (D) Residue-specific analysis of the total fraction of contacts between Ser303 or Phe303 and their interaction partners within GALC. (E) Structural representation of the hydrogen bond (indicated by a black dashed line) between the hydroxyl group of Ser303 and backbone carbonyl group of Gly57 (black dashed line). This interaction is present only in wild-type GALC (see Fig. S8a). Hydrogen-bond occurrence was measured for each simulation run of the wild type and the GALC variant with Ser303Phe. (F) Structural representation of the hydrogen bonds (indicated by black dashed lines) between the amino group in the backbone of Ser272 or the side chain of Ser272 and the backbone carbonyl group of Ser303/Phe303. Hydrogen-bond occurrence was measured as in (E). (A, B, and D to F) Mean values are shown as bar graphs ± standard error of the mean (SEM) with circles representing individual simulation runs (*n* = 4). Statistical testing was performed in GraphPad Prism (V10) using (A, B, and E) an unpaired *t* test, (D) 2-way analysis of variance (ANOVA) with Šídák’s multiple comparisons test, or (F) one-way ANOVA with Šídák’s multiple comparisons test (*P < 0.05; **P < 0.01; ****P < 0.0001).

The Ser303Phe missense mutation, like Gly59Arg, is located within one of the β-strands that constitute the TIM-barrel motif (Fig. 1A). Similar to the other GALC variants, Ser303Phe induced distinct structural alterations compared to the wild type. The backbone fluctuations of Phe303 were higher than those of Ser303 in wild-type GALC (Fig. 6A). Due to the bulkier and hydrophobic phenylalanine side chain replacing the smaller and polar serine side chain, the mutation Ser303Phe introduced a significantly higher number of intramolecular contacts (Fig. 6B). The detailed analysis of the total fraction of contacts of the residue at position 303 showed that Phe303 has significantly higher fractions of total contacts with Asp56, Gly59, Ile97, Met147, Trp271, Ser272, Ser273, Thr304, and Ile305 (Fig. 6C and D). The variant Ser303Phe demonstrated an altered hydrogen-bonding behavior: The hydrogen bond between the Ser303 side chain and the backbone of Gly57 was completely lost (Fig. 6E and Fig. S8a) due to the nonpolar nature of the phenylalanine side chain. Furthermore, the hydrogen-bond network between Ser303 and Ser272 in the neighboring β-strand was changed: The hydrogen bond with the backbone was significantly weakened, whereas the hydrogen bond with the hydroxyl group in the side chain of Ser272 was slightly more stable in the GALC variant Ser303Phe (Fig. 6F and Fig. S8b). Moreover, the backbone of residue Phe303 in the GALC variant Ser303Phe exhibited a more stable hydrogen bond with the side chain of the directly neighboring Thr304 as evidenced by slightly higher hydrogen-bond occurrence rates (Fig. S8c). Thus, comparable to the Ser68Phe variant, the Ser303Phe variant showed reduced hydrogen bonding that might result in higher protein flexibility (Fig. 2).

### Influence of mutations on the active-site architecture of GALC

Missense mutations in enzymes can impair activity through diverse mechanisms, including disruption of active-site geometry, altered substrate binding, or compromised catalytic residues, resulting in reduced function compared to the wild-type enzyme. Accordingly, we next investigated whether individual GALC variants significantly alter the structure and thereby potentially also the function of the active site, contributing to KD pathogenesis. Elucidating how specific amino acid substitutions reshape GALC’s active-site architecture remains crucial for decoding the genotype–enzyme dysfunction relationship and pinpointing therapeutically targetable sites.

#### Volume of the active-site pocket

**Fig. 7.**
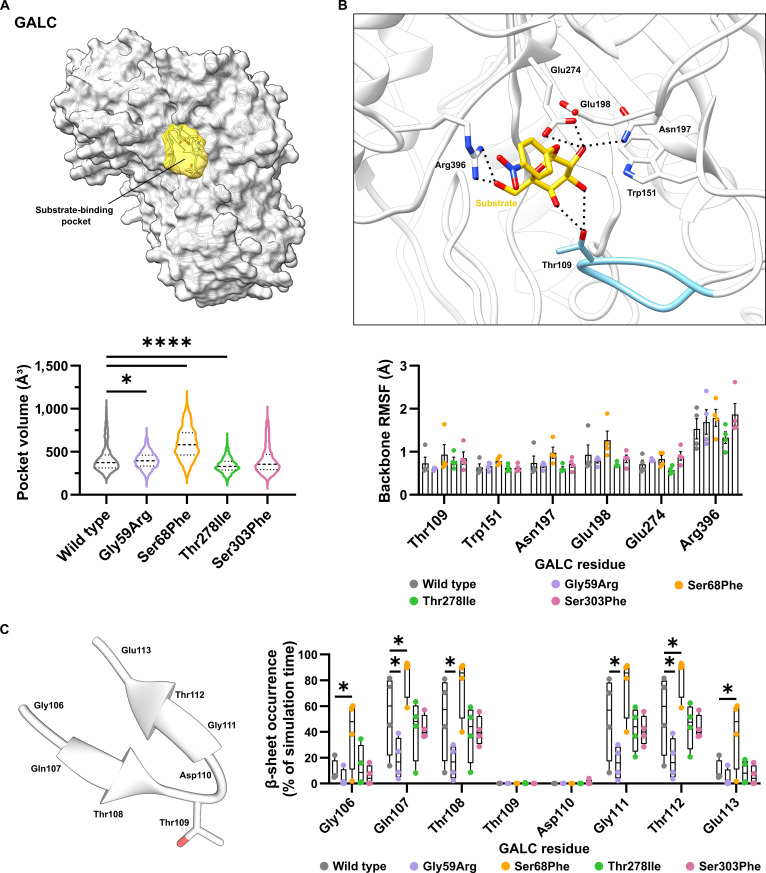
Influence of mutations on the active-site architecture of β-galactocerebrosidase (GALC). (A) Structural representation of GALC with highlighted substrate-binding pocket (yellow; upper panel) and the volume of the substrate-binding pocket of the wild-type GALC protein and each GALC variant summarized over all 4 simulation runs (dashed lines: median; dotted lines: quartiles; lower panel). (B) Structural representation and root-mean-square fluctuation (RMSF) values of the substrate-binding residues (Thr109, Trp151, Asn197, and Arg396) and active-site residues (Glu198 and Glu274). The loop region Gly106 to Glu113 (around the substrate-binding Thr109) is colored blue to highlight the region described in (C). The black dashed lines indicate hydrogen bonds (upper panel). Mean values ± standard error of the mean (SEM) are shown as bar graphs with circles representing individual simulation runs (*n* = 4; lower panel). (C) Structural representation of the β-hairpin Gly106 to Glu113 and β-sheet occurrence of the region Gly106 to Glu113. Values of the single runs are shown as box plots (min to max with the mean as a horizontal line). (A to C) Statistical testing was performed in GraphPad Prism (V10) using (A) one-way analysis of variance (ANOVA) with Dunnett’s multiple comparisons test or (B and C) 2-way ANOVA with (B) Dunnett’s or (C) Šídák’s multiple comparisons test (*P < 0.05; ****P < 0.0001).

Analysis of active-site pocket volumes across 1,000 evenly distributed snapshots (250 from each of the 4 simulation runs) revealed significant differences between 3 of the 4 missense variants (Gly59Arg, Ser68Phe, and Thr278Ile) and the wild type (Fig. 7A). Enzymatic pocket sizes intrinsically fluctuate due to so-called breathing motions, which are essential for catalytic function as they permit conformational flexibility, substrate accommodation, and efficient turnover. In the wild type, pocket volumes ranged from 179.0 to 1,056.8 Å^3^, with the most values between 200 and 600 Å^3^. Comparison of the variants with the wild type revealed 2 distinct trends. First, variants Gly59Arg and Thr278Ile exhibited reduced maximal pocket volumes (Gly59Arg and Thr278Ile not exceeding 750 Å^3^) compared to the wild type, while their median values remained comparable. While the GALC wild type has a pocket volume greater than 600 Å^3^ in 13.2% of the simulation time, this is reduced to 3.3% and 0.9% in the Gly59Arg and Thr278Ile variants, respectively. Second, the Ser68Phe variant showed a shift toward larger minimal and maximal volumes (209.2 and 1,133.3 Å^3^, respectively) and a notably higher median value (582.6 Å^3^ [Ser68Phe] *vs.* 373.0 Å^3^ [wild type]). Also, volumes exceeding 600 Å^3^ occurred in the GALC Ser68Phe variant considerably more frequently than in the wild type. Thus, Ser68Phe seems to remain in a more open configuration, which might negatively affect the enzymatic activity of the variant. In contrast, Gly59Arg and Thr278Ile remain in rather closed conformations, which might impede the entering of the substrate into the binding pocket.

#### Flexibility of residues at the active/binding site

A plausible explanation for the impaired enzymatic function of GALC, leading to the manifestation of KD, can also be that mutations alter the fluctuations of substrate-binding or catalytic residues. However, the MD simulations revealed that the substrate-binding residues Thr109, Trp151, Asn197, and Arg396, as well as the catalytic residues Glu198 and Glu274, showed no significant differences in fluctuation compared to the wild type (Fig. 7B). Among the 4 analyzed variants, only the Ser68Phe variant exhibited slightly increased fluctuations for all of the substrate-binding or catalytic residues relative to the wild type. In contrast, the Thr278Ile variant resulted in a minor decrease in the backbone RMSF values for most of the substrate-binding or catalytic residues. As these changes in the backbone RMSF values, however, were not significant, additional structural or dynamic factors may contribute to the pathogenicity of these variants.

#### Influence on the β-hairpin structure adjacent to the substrate-binding Thr109

In a recent study of the GALC Thr112Ala variant, a reduction in the stability of the β-hairpin in the region of residues Gly106 to Glu113 was observed for the GALC Thr112Ala variant compared to that of the wild-type enzyme [15]. This structural element flanks the substrate-binding residue Thr109 (Fig. 7B and C), whose orthologous position in murine GALC interacts with the substrate (PDB ID: 4CCC [21]), the reaction intermediate (PDB ID: 4CCD [21]), and the product (PDB ID: 4CCE [21]) via 2 hydrogen bonds. Although the β-hairpin is not resolved in the murine GALC crystal structure (PDB ID: 4CCC [21]), our data support the notion that it forms transiently and contributes to local stabilization of the catalytic region. To evaluate its dynamic behavior, we quantified the occurrence of β-sheet formation among residues Gly106 to Glu113 across the MD simulation trajectories. The wild-type enzyme showed broad fluctuations, consistent with a highly flexible, short-lived β-hairpin (Fig. 7C). The GALC variants Gly59Arg, Thr278Ile, and Ser303Phe exhibited markedly reduced β-sheet formation frequencies, suggesting local destabilization and impaired structural breathing motions near the active site. In contrast, the Ser68Phe variant displayed an increased persistence of the β-hairpin structure, indicating a more rigid configuration that could restrict conformational adaptability during catalysis. Together, these observations highlight the sensitivity of the β-hairpin region to single-residue substitutions and suggest that altered structural stability in this loop may modulate catalytic efficiency in GALC.

#### Influence on the structural stability of the TIM barrel

**Fig. 8.**
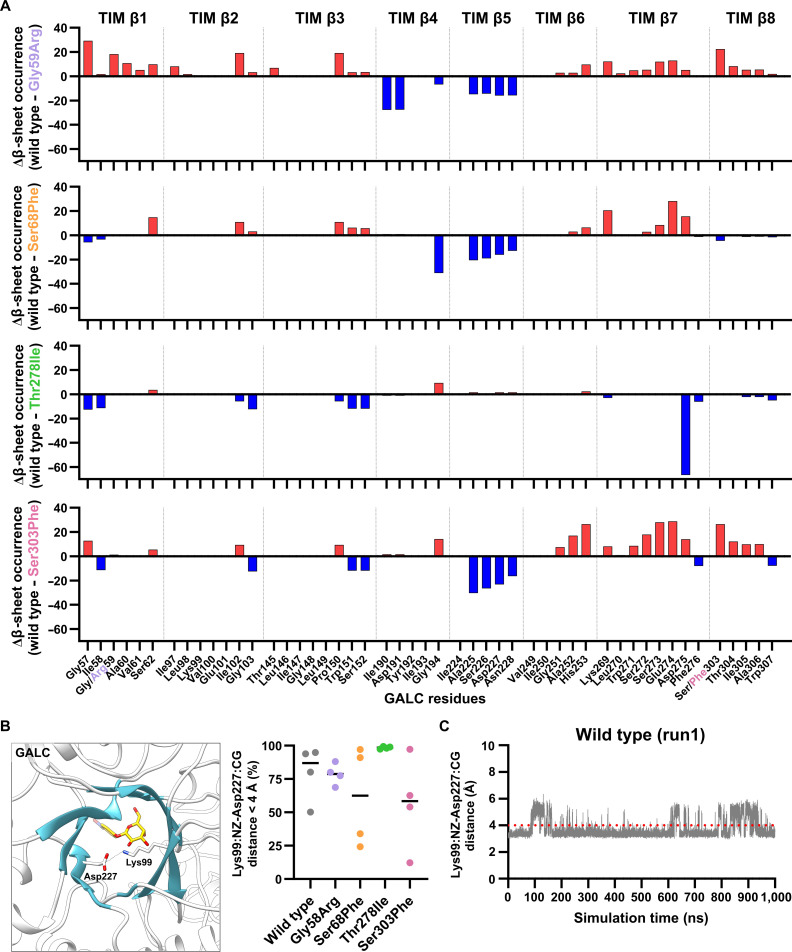
Influence on the structural stability of the inner β-barrel of the triosephosphate isomerase (TIM) barrel. (A) β-sheet occurrence of all 8 β-strands (TIM β1 to TIM β8) forming the inner β-barrel of the central TIM barrel calculated for each residue compared with the corresponding residue in the wild-type β-galactocerebrosidase (GALC; Δβ-sheet occurrence). Blue bars indicate a higher β-sheet occurrence in the GALC variant compared to the wild type, whereas red bars indicate a lower β-strand stability. All plots with the original data for all runs of the wild-type GALC protein and all GALC variants can be found in Figs. S9 to S12. (B) Structural representation of the salt bridge (indicated by a black dashed line) between the side-chain atoms of Lys99:NZ and Asp227:CG. For better structural orientation, the inner β-barrel of the TIM barrel is colored blue and a substrate molecule was inserted to indicate the active site. Salt bridge occurrence (distance between both atoms < 4 Å) was measured as percentage over the course of the simulation for each simulation run of the wild type and the GALC variants (mean values are shown in color; the black line indicates the mean of all 4 simulation runs). (C) Time-resolved distance plot for one molecular dynamics (MD) simulation run of the wild-type GALC protein in order to describe the distance between the Lys99:NZ atom and the Asp227:CG atom. The red dotted lines indicate a distance of 4 Å, and if the measured distance is below this value, a salt bridge is likely present. All plots for all runs of the wild-type GALC protein and all GALC variants can be found in Figs. S13 and S14.

Human GALC features a central TIM barrel that forms the core catalytic scaffold of the enzyme and dominates its overall architecture. The TIM barrel spans residues 57 to 353 and consists of 8 parallel β-strands (Figs. 1A and 8A) surrounded by 8 α-helices, with the active site formed primarily by the long loops on the C-terminal face of the barrel that shape the substrate-binding pocket. These loops host key catalytic and substrate-recognition residues, thereby coupling the conserved TIM-barrel fold to the specific chemistry of galactosylceramide hydrolysis in KD. Extensive interfaces between the TIM barrel and the flanking β-sandwich and lectin domains rigidify the relative domain arrangement, suggesting that the barrel not only provides the catalytic platform but also orchestrates interdomain contacts that stabilize the active-site geometry and facilitate cooperative contributions of distal loops to substrate binding.

The stability of the inner β-barrel of the TIM barrel, which consists of 8 parallel β-strands and harbors the substrate-binding Trp151 and the catalytic Glu274, was described by the analysis of the β-strand occurrence for each of the residues in the inner β-barrel (Fig. 8A and Figs. S9 to S12). In the GALC variant Gly59Arg, the β-strands TIM β4 and TIM β5 had higher β-sheet propensities, while all of the other 6 β-strands harbored residues with lower propensities compared with the wild type. In particular, the mutation site Arg59 itself and residues interacting with Arg59 and their direct neighbors (for instance, Gly57-Ser62, Lys269-Asp275, and Ser303-Trp307) showed lower β-sheet occurrence compared to the wild type (Fig. 8A and Fig. S9). The GALC variants Ser68Phe and Ser303Phe showed very similar results (Fig. 8A and Figs. S10 and S12) compared with the GALC variant Gly59Arg. In contrast, in the GALC Thr278Ile variant, the measured values for nearly all residues in the inner β-barrel were close to those of the wild type, with only Asp275 exhibiting a significantly higher β-sheet propensity (Fig. 8A and Fig. S11). This is noteworthy, as Asp275 is the residue directly adjacent to the catalytic residue Glu274, which in this GALC variant also displayed slightly reduced backbone fluctuations compared to the wild type (cf. Fig. 7B). Thus, this localized stabilization of the β-strand likely anchors the active-site geometry. This finding is consistent with the markedly constricted substrate-binding pocket volume observed in this GALC variant (Fig. 7A).

Furthermore, TIM-barrel proteins are known to stabilize their inner β-barrel through one or more salt bridges [45], which are structure-stabilizing electrostatic interactions between positively and negatively charged amino acid side chains. In GALC, such a salt bridge is formed between Lys99 (β-strand TIM β2) and Asp227 (β-strand TIM β5, Fig. 8B and C and Figs. S13 and S14). While this Lys99-Asp227 salt bridge interaction remained highly stable in the wild-type GALC, it occurred less frequently in the Gly59Arg, Ser68Phe, and Ser303Phe variants. This observation correlates with the result of the β-sheet analysis of the β-strand TIM β5, which was more stable in all these 3 GALC variants compared with the wild type (Fig. 8A). The GALC variant Thr278Ile maintained this Lys99-Asp227 salt bridge for nearly the full time of the MD simulations (Fig. 8B and Fig. S14a) and showed a nearly unchanged β-sheet propensity for β-strand TIM β5 compared to the wild-type GALC. These results suggest that the stability of the Lys99-Asp227 salt bridge negatively correlates with an enhanced stabilization of the β-strand TIM β5 in the inner β-barrel of the TIM barrel.

Taken together, our MD simulations demonstrate that the 4 investigated missense variants alter to different extents the conformational flexibility of human GALC and modulate intramolecular contacts between different amino acids, for instance, by changing the intramolecular network of hydrogen bonds and other stabilizing interactions (e.g., cation–π interactions). These perturbations propagate toward the substrate-binding region through dynamic coupling of secondary-structure elements, providing a molecular rationale for the experimentally observed changes in catalytic efficiency under lysosomal conditions. Notably, the 4 investigated variants do not affect the structure of the enzyme in a uniform manner, but each induces distinct structural rearrangements and interaction patterns, highlighting multiple molecular routes by which local alterations might influence the enzymatic function of GALC. However, our structure-based explanations for the reduced enzymatic activity of GALC have to be verified on an experimental level.

## Discussion

KD is a lysosomal storage disorder caused by mutations in the *GALC* gene. These mutations reduce the activity of the lysosomal hydrolase GALC, leading to an accumulation of psychosine. This toxic metabolite triggers apoptosis in oligodendrocytes and Schwann cells. Although more than 140 GALC variants have been identified [46], the specific impact of many individual variants on enzyme activity and thus their role in disease manifestation remain unclear.

The infantile form of KD is often diagnosed during newborn screening protocols. Initial testing for KD involves measurement of GALC enzymatic activity in blood leukocytes or skin fibroblasts. Low GALC activity alone, however, is insufficient for diagnosis. Therefore, genetic testing is performed for the identification of disease-causing mutations. However, only few GALC variants are clearly associated with disease progression, whereas the impact of many variants is unclear. As the disease is caused by increasing levels of the toxic metabolite psychosine, measurement of psychosine in blood or cerebral spinal fluid is an emerging biomarker for diagnosis as well as monitoring of disease progression. Further, imaging, such as computed tomographic scans or magnetic resonance imaging, can aid in the diagnosis. However, therapies except for hematopoietic stem cell transplantation are missing and the development of new therapeutic approaches might benefit from structural insights into how individual variants affect enzyme activity. In this study, we selected 4 variants associated with the infantile form of KD in heterozygous or homozygous patients [16–18]. These 4 variants have in common that the missense mutations are located outside the substrate-binding region and are not directly involved in catalytic function. Consequently, it is not yet understood how these variants impair GALC activity.

The aim of this study was to identify a molecular explanation for the pathogenic nature of these variants, which may aid in diagnosing patients with compound-heterozygous GALC variants and support the development of new therapeutic strategies. Further, our results might also help in the diagnosis of late- or adult-onset forms, which are more difficult to diagnose due to slower progression and overlap with other neurodegenerative diseases such as multiple sclerosis. Moreover, due to advances in molecular diagnostics, attenuated forms of lysosomal storage diseases including KD with milder phenotypes are diagnosed more often [47,48]. MD simulations of unclear variants could complement results of biochemical assays and biomarkers, such as GALC activity and psychosine level, respectively. Thereby, the time until diagnosis could be reduced, which often takes years for patients with adult-onset or attenuated forms.

To address this question, we applied a range of *in silico* approaches, including AlphaFold-predicted models of GALC, all-atom MD simulations with fixed protonation states appropriate for the lysosomal environment (pH 4.5), and structural bioinformatics analyses. Our results show that these variants alter protein flexibility, disrupt the hydrogen-bond network, affect the stability of secondary structural elements, and change the accessibility of the substrate-binding pocket under lysosomal conditions.

We identified shared effects among 3 of the 4 variants, as they all affected the secondary-structure motifs of the TIM barrel in a similar manner. The TIM barrel itself is stabilized by a salt bridge between Lys99 and Asp227. We showed that the likelihood of this interaction is affected by each mutation and alters the frequency of β-sheet formation within the TIM motif. As Trp151 is part of both the binding site and the TIM barrel (cf. TIM β3 in Fig. 8A) and Glu274 is part of the active site as well as the TIM barrel, structural changes in the TIM barrel may lead to altered enzymatic behavior. In patients with 3-hydroxy-3-methylglutaric aciduria, the disease can be caused by mutations affecting the TIM barrel of the enzyme 3-hydroxy-3-methylglutaryl-coenzyme A lyase [49]. The TIM-barrel motif is not exclusive to GALC or 3-hydroxy-3-methylglutaryl-coenzyme A lyase; instead, it is shared by at least 15 distinct enzyme families that employ this structural framework to generate the appropriate active-site geometry. Comparative analyses of sequence and structure indicate that many TIM-barrel enzymes are evolutionarily related [50]. Glycoside hydrolases, the enzyme family to which GALC belongs, represent one of these functionally diverse groups that typically adopt the TIM-barrel fold [51,52]. The relevance of the TIM-barrel motif is further underscored by TIM itself. This enzyme, after which the TIM-barrel fold is named, harbors several disease-causing mutations within the TIM motif [53,54]. Such mutations impair structural stability or catalytic efficiency and result in clinically relevant enzyme deficiencies, highlighting the critical functional role of the TIM-barrel architecture. Disruption of this structural framework therefore provides a strong indicator of why a given mutation leads to a loss of enzymatic function. Thus, our methodological framework might be expanded to other enzymes with TIM-barrel fold.

Further, the region surrounding the substrate-binding residue Thr109 seems to be important. All 4 of the investigated variants alter the propensity of the loop surrounding Thr109 to form a β-hairpin. As a consequence, the loop becomes either less flexible due to increased β-sheet stability (as observed for Ser68Phe) or excessively flexible due to reduced β-sheet formation (as seen in Gly59Arg, Thr278Ile, and Ser303Phe; cf. Fig. 7C), which may result in a different substrate pocket geometry evidenced by a significantly altered volume of the substrate-binding pocket for 3 of the 4 investigated mutations: increasing in the case of Ser68Phe and decreasing maximal volumes in the case of Gly59Arg and Thr278Ile (cf. Fig. 7A). The altered structural stability of the Gly106 to Glu113 region, with the substrate-binding residue Thr109, appears to be a key contributing factor to changes in the volume of the substrate-binding pocket and may lead to a decrease in enzymatic activity, as conformations that allow substrate entry into the pocket or product release from the pocket may be adopted less frequently. Thus, our results might provide an atomic-level explanation of how some mutations in GALC contribute to the development of KD. However, this model of how the stability of a β-hairpin influences the enzymatic activity of GALC has to be proven experimentally but would align with findings from an *in silico* study of the Thr112Ala variant using homology modeling and all-atom MD simulations with both cytosolic (pH 7.0) and lysosomal (pH 4.5) protonation states [15]. In that study, the Thr112Ala mutation was also shown to alter the pocket’s overall volume and therefore potentially influence substrate entry into the binding pocket. Remarkably, although the 4 variants described here and the Thr112Ala substitution differ in their structural contexts and specific local effects, they all ultimately result in a comparable functional outcome: a reduction or complete loss of enzymatic activity. While not all variants substantially alter the size or geometry of the substrate-binding pocket, several induce distinct perturbations in the β-sheet region surrounding Thr109 (either stabilizing or destabilizing it). These MD-simulation-based findings might suggest that local structural distortions within or near the substrate-recognition region, even when mechanistically diverse, converge to disrupt catalytic function and thus underlie the early infantile form of KD.

It should be noted, however, that this represents only one of several possible mechanisms by which GALC functionality can be reduced. The degradation of sphingolipids requires the interaction of GALC with saposin A, and mutations that impair this interaction could therefore also give rise to KD [55]. While the mutation sites described here are not part of the proposed interface between saposin A and GALC, the changed pocket geometry might prevent the delivery of hydrophobic substrates such as psychosine to the active site. As shown in a crystal structure of a heterotetramer of 2 GALC and 2 saposin A molecules [55], saposin A delivers the hydrophilic glycosyl head groups to the active site of GALC while containing the hydrophobic acyl chains in a hydrophobic cavity between the 2 saposin A dimers [55]. Changes in the geometry of the binding pocket might block the entry of the substrate. However, structures of GALC and saposin A with bound substrates do not exist yet, whereby it is not possible to test this hypothesis with our methods.

Additional mechanisms have been described by which mutations in GALC can lead to KD independently of direct impairments in enzymatic activity. The Leu629Arg variant causes defective trafficking of GALC, resulting in retention of the protein in the endoplasmic reticulum due to failure of lysosomal transport [56]. Similarly, the Asp528Asn variant leads to disease manifestation through protein misfolding caused by the introduction of a second N-glycosylation site [56]. Of particular interest is the fact that a strategy to restore the function of the GALC variant Asp528Asn has already been described. In this context, α-lobeline, a relatively weak inhibitor of GALC, acts as a pharmacological chaperone and has been shown to rescue the function of this variant [56]. To date, however, it has not been demonstrated that other variants causing comparable structural alterations can likewise be functionally restored. Nevertheless, this represents a highly promising approach for the development of therapeutic GALC activators.

In general, variants could be classified according to their impact on enzyme structure and catalytic behavior, allowing for the development of mutation-class-specific pharmacological interventions. A comparable concept is well established for the genetic disease cystic fibrosis, where different modulators of protein function are available that can restore the activity of specific variants depending on the mutation class [57]. It should be noted, however, that cystic fibrosis is caused by a defect in an ion channel rather than in an enzyme with catalytic activity.

For GALC, the x-ray crystal structures of murine GALC in complex with the specific inhibitor galacto-noeurostegine have been solved at both neutral and lysosomal pH, revealing a potential allosteric binding site [58]. This site could potentially be targeted through the design of pharmacological chaperones; however, its exact nature remains unclear and requires further investigation. In addition, activators have already been identified for β-glucocerebrosidase, a lysosomal enzyme that is functionally and structurally closely related to GALC. These activators bind to the protein surface outside the active site and enhance enzymatic activity [12]. Our mutational analysis further enabled us to identify regions of the protein whose dynamic behavior is altered by individual mutations, suggesting that these regions may serve as potential targets for allosteric activation. A task for future studies could be to identify additional targets and to characterize other mechanisms by which GALC variants cause KD. In particular, the interaction with saposin A warrants further investigation.

## Conclusions

The MD simulations performed showed that all 4 variants have effects on the protein structure and that they go far beyond the predictable effects obtained from homology modeling. The most striking effect is that the Ser68Phe missense variant induces the largest structural perturbation within the binding pocket, which appears enlarged in the MD simulations. This enlargement might impair substrate interactions with binding pocket residues, thereby giving a structure-based explanation for the reduced enzymatic activity. Additionally, the stability of the β-hairpin surrounding the substrate-binding residue Thr109 is increased by Ser68Phe or decreased by Gly59Arg, Thr278Ile, and Ser303Phe relative to the wild-type enzyme. Therefore, the results suggest that if the structural stability of the region around Thr109 is higher, the loop will be too rigid and substrates will find it more difficult to enter the binding pocket, whereas if the average stability is lower, the substrate binding will not be strong enough. Either of these can lead to a longer time between the conversion of 2 substrate molecules and therefore a lower activity measured in the experiment. However, our structure-based explanations have to be verified with MD simulations of GALC with bound ligand as well as with wet lab experiments.

## Data Availability

All data needed to evaluate the conclusions of this study are available in the paper and the Supplementary Materials. Further data are available from the corresponding author upon reasonable request.

## References

[1] Platt FM, d’Azzo A, Davidson BL, Neufeld EF, Tifft CJ. Lysosomal storage diseases. Nat Rev Dis Primers. 2018;4(1):27. 30275469 10.1038/s41572-018-0025-4

[2] Li Y, Xu Y, Benitez BA, Nagree MS, Dearborn JT, Jiang X, Guzman MA, Woloszynek JC, Giaramita A, Yip BK, et al. Genetic ablation of acid ceramidase in Krabbe disease confirms the psychosine hypothesis and identifies a new therapeutic target. Proc Natl Acad Sci USA. 2019;116(40):20097–20103. 31527255 10.1073/pnas.1912108116 PMC6778236

[3] Nagara H, Ogawa H, Sato Y, Kobayashi T, Suzuki K. The twitcher mouse: Degeneration of oligodendrocytes in vitro. Brain Res. 1986;391(1):79–84. 3513905 10.1016/0165-3806(86)90009-x

[4] Yoon IC, Bascou NA, Poe MD, Szabolcs P, Escolar ML. Long-term neurodevelopmental outcomes of hematopoietic stem cell transplantation for late-infantile Krabbe disease. Blood. 2021;137(13):1719–1730. 33150395 10.1182/blood.2020005477 PMC8020262

[5] Babcock MC, Mikulka CR, Wang B, Chandriani S, Chandra S, Xu Y, Webster K, Feng Y, Nelvagal HR, Giaramita A, et al. Substrate reduction therapy for Krabbe disease and metachromatic leukodystrophy using a novel ceramide galactosyltransferase inhibitor. Sci Rep. 2021;11(1):14486. 34262084 10.1038/s41598-021-93601-1 PMC8280112

[6] Sands SA, LeVine SM. Substrate reduction therapy for Krabbe’s disease. J Neurosci Res. 2016;94(11):1261–1272. 27638608 10.1002/jnr.23791

[7] Biswas S, Biesiada H, Williams TD, LeVine SM. Substrate reduction intervention by l-cycloserine in twitcher mice (globoid cell leukodystrophy) on a B6;CAST/Ei background. Neurosci Lett. 2003;347(1):33–36. 12865135 10.1016/s0304-3940(03)00633-5

[8] Hordeaux J, Jeffrey BA, Jian J, Choudhury GR, Michalson K, Mitchell TW, Buza EL, Chichester J, Dyer C, Bagel J, et al. Efficacy and safety of a Krabbe disease gene therapy. Hum Gene Ther. 2022;33(9–10):499–517. 35333110 10.1089/hum.2021.245 PMC9142772

[9] Bradbury AM, Bagel JH, Nguyen D, Lykken EA, Salvador JP, Jiang X, Swain GP, Assenmacher CA, Hendricks IJ, Miyadera K, et al. Krabbe disease successfully treated via monotherapy of intrathecal gene therapy. J Clin Invest. 2020;130(9):4906–4920. 32773406 10.1172/JCI133953 PMC7456224

[10] Bradbury AM, Bongarzone ER, Sands MS. Krabbe disease: New hope for an old disease. Neurosci Lett. 2021;752: Article 135841. 33766733 10.1016/j.neulet.2021.135841 PMC8802533

[11] Benz J, Rufer AC, Huber S, Ehler A, Hug M, Topp A, Guba W, Hofmann EC, Jagasia R, Rodríguez Sarmiento RM. Novel β-glucocerebrosidase activators that bind to a new pocket at a dimer Interface and induce dimerization. Angew Chem Int Ed Engl. 2021;60(10):5436–5442. 33238058 10.1002/anie.202013890

[12] Schulze M-SED, Scholz D, Jnoff E, Hall A, Melin J, Sands ZA, Rodriguez E, Andre VM. Identification of ß-glucocerebrosidase activators for glucosylceramide hydrolysis. ChemMedChem. 2024;19(7): Article e202300548. 38381042 10.1002/cmdc.202300548

[13] Nashabat M, Al-Khenaizan S, Alfadhel M. Report of a case that expands the phenotype of infantile Krabbe disease. Am J Case Rep. 2019;20:643–646. 31053700 10.12659/AJCR.914275 PMC6512756

[14] Mächtel R, Dobert J-P, Hehr U, Weiss A, Kettwig M, Laugwitz L, Groeschel S, Schmidt M, Arnold P, Regensburger M, et al. Late-onset Krabbe disease presenting as spastic paraplegia—Implications of GCase and CTSB/D. Ann Clin Transl Neurol. 2024;11(7):1715–1731. 38837642 10.1002/acn3.52078 PMC11251474

[15] Heger L, Ankermann P, Socher E. Molecular characterization of the GALC mutation Thr112Ala causing Krabbe disease. Int J Mol Sci. 2025;26(17). 10.3390/ijms26178647 PMC12429000 40943566

[16] Fu L, Inui K, Nishigaki T, Tatsumi N, Tsukamoto H, Kokubu C, Muramatsu T, Okada S. Molecular heterogeneity of Krabbe disease. J Inherit Metab Dis. 1999;22(2):155–162. 10234611 10.1023/a:1005449919660

[17] Wenger DA, Rafi MA, Luzi P. Molecular genetics of Krabbe disease (globoid cell leukodystrophy): Diagnostic and clinical implications. Hum Mutat. 1997;10(4):268–279. 9338580 10.1002/(SICI)1098-1004(1997)10:4<268::AID-HUMU2>3.0.CO;2-D

[18] Wenger DA, Luzi P, Rafi MA. Advances in the diagnosis and treatment of Krabbe disease. Int J Neonatal Screen. 2021;7(3):57. 34449528 10.3390/ijns7030057 PMC8396024

[19] Jumper J, Evans R, Pritzel A, Green T, Figurnov M, Ronneberger O, Tunyasuvunakool K, Bates R, Žídek A, Potapenko A, et al. Highly accurate protein structure prediction with AlphaFold. Nature. 2021;596(7873):583–589. 34265844 10.1038/s41586-021-03819-2 PMC8371605

[20] Deane JE, Graham SC, Kim NN, Stein PE, McNair R, Cachón-González MB, Cox TM, Read RJ. Insights into Krabbe disease from structures of galactocerebrosidase. Proc Natl Acad Sci USA. 2011;108(37):15169–15173. 21876145 10.1073/pnas.1105639108 PMC3174575

[21] Hill CH, Graham SC, Read RJ, Deane JE. Structural snapshots illustrate the catalytic cycle of β-galactocerebrosidase, the defective enzyme in Krabbe disease. Proc Natl Acad Sci USA. 2013;110(51):20479–20484. 24297913 10.1073/pnas.1311990110 PMC3870757

[22] Jurrus E, Engel D, Star K, Monson K, Brandi J, Felberg LE, Brookes DH, Wilson L, Chen J, Liles K, et al. Improvements to the APBS biomolecular solvation software suite. Protein Sci. 2018;27(1):112–128. 28836357 10.1002/pro.3280 PMC5734301

[23] Socher E, Sticht H, Horn AHC. The conformational stability of nonfibrillar amyloid-β peptide oligomers critically depends on the C-terminal peptide length. ACS Chem Neurosci. 2014;5(3):161–167. 24494584 10.1021/cn400208r PMC3963130

[24] Socher E, Conrad M, Heger L, Paulsen F, Sticht H, Zunke F, Arnold P. Mutations in the B.1.1.7 SARS-CoV-2 spike protein reduce receptor-binding affinity and induce a flexible link to the fusion peptide. Biomedicine. 2021;9(5):525. 10.3390/biomedicines9050525 PMC8151884 34066729

[25] Socher E, Conrad M, Heger L, Paulsen F, Sticht H, Zunke F, Arnold P. Computational decomposition reveals reshaping of the SARS-CoV-2-ACE2 interface among viral variants expressing the N501Y mutation. J Cell Biochem. 2021;122(12):1863–1872. 34516024 10.1002/jcb.30142

[26] Socher E, Heger L, Paulsen F, Zunke F, Arnold P. Molecular dynamics simulations of the delta and omicron SARS-CoV-2 spike - ACE2 complexes reveal distinct changes between both variants. Comput Struct Biotechnol J. 2022;20:1168–1176. 35251533 10.1016/j.csbj.2022.02.015 PMC8881326

[27] Case DA, Aktulga HM, Belfon K, Ben-Shalom IY, Berryman JT, Brozell SR, et al. Amber 2023.

[28] Maier JA, Martinez C, Kasavajhala K, Wickstrom L, Hauser KE, Simmerling C. ff14SB: Improving the accuracy of protein side chain and backbone parameters from ff99SB. J Chem Theory Comput. 2015;11(8):3696–3713. 26574453 10.1021/acs.jctc.5b00255 PMC4821407

[29] Jorgensen WL, Chandrasekhar J, Madura JD, Impey RW, Klein ML. Comparison of simple potential functions for simulating liquid water. J Chem Phys. 1983;79(2):926–935.

[30] Götz AW, Williamson MJ, Xu D, Poole D, Le Grand S, Walker RC. Routine microsecond molecular dynamics simulations with AMBER on GPUs. 1. Generalized Born. J Chem Theory Comput. 2012;8(5):1542–1555. 22582031 10.1021/ct200909j PMC3348677

[31] Salomon-Ferrer R, Götz AW, Poole D, Le Grand S, Walker RC. Routine microsecond molecular dynamics simulations with AMBER on GPUs. 2. Explicit solvent particle mesh Ewald. J Chem Theory Comput. 2013;9(9):3878–3888. 26592383 10.1021/ct400314y

[32] Le Grand S, Götz AW, Walker RC. SPFP: Speed without compromise—A mixed precision model for GPU accelerated molecular dynamics simulations. Comput Phys Commun. 2013;184(2):374–380.

[33] Berendsen HJC, Postma JPM, van Gunsteren WF, DiNola A, Haak JR. Molecular dynamics with coupling to an external bath. J Chem Phys. 1984;81(8):3684–3690.

[34] Ryckaert J-P, Ciccotti G, Berendsen HJ. Numerical integration of the cartesian equations of motion of a system with constraints: Molecular dynamics of *n*-alkanes. J Comput Phys. 1977;23(3):327–341.

[35] Roe DR, Cheatham TE. PTRAJ and CPPTRAJ: Software for processing and analysis of molecular dynamics trajectory data. J Chem Theory Comput. 2013;9(7):3084–3095. 26583988 10.1021/ct400341p

[36] Ma JC, Dougherty DA. The cation−π interaction. Chem Rev. 1997;97(5):1303–1324. 11851453 10.1021/cr9603744

[37] Durrant JD, Votapka L, Sørensen J, Amaro RE. POVME 2.0: An enhanced tool for determining pocket shape and volume characteristics. J Chem Theory Comput. 2014;10(11):5047–5056. 25400521 10.1021/ct500381c PMC4230373

[38] Guerra J, Belleri M, Paiardi G, Tobia C, Capoferri D, Corli M, Scalvini E, Ghirimoldi M, Manfredi M, Wade RC, et al. Impact of an irreversible β-galactosylceramidase inhibitor on the lipid profile of zebrafish embryos. Comput Struct Biotechnol J. 2024;23:1397–1407. 38596316 10.1016/j.csbj.2024.03.023 PMC11002810

[39] Pettersen EF, Goddard TD, Huang CC, Couch GS, Greenblatt DM, Meng EC, Ferrin TE. UCSF Chimera—A visualization system for exploratory research and analysis. J Comput Chem. 2004;25(13):1605–1612. 15264254 10.1002/jcc.20084

[40] Goddard TD, Huang CC, Meng EC, Pettersen EF, Couch GS, Morris JH, Ferrin TE. UCSF ChimeraX: Meeting modern challenges in visualization and analysis. Protein Sci. 2017;27(1):14–25. 28710774 10.1002/pro.3235 PMC5734306

[41] Pettersen EF, Goddard TD, Huang CC, Meng EC, Couch GS, Croll TI, Morris JH, Ferrin TE. UCSF ChimeraX: Structure visualization for researchers, educators, and developers. Protein Sci. 2021;30(1):70–82. 32881101 10.1002/pro.3943 PMC7737788

[42] Meng EC, Goddard TD, Pettersen EF, Couch GS, Pearson ZJ, Morris JH, Ferrin TE. UCSF ChimeraX: Tools for structure building and analysis. Protein Sci. 2023;32(11): Article e4792. 37774136 10.1002/pro.4792 PMC10588335

[43] Gouet P, Robert X, Courcelle E. ESPript/ENDscript: Extracting and rendering sequence and 3D information from atomic structures of proteins. Nucleic Acids Res. 2003;31(13):3320–3323. 12824317 10.1093/nar/gkg556 PMC168963

[44] Sievers F, Wilm A, Dineen D, Gibson TJ, Karplus K, Li W, Lopez R, William HM, Remmert M, Söding J, et al. Fast, scalable generation of high-quality protein multiple sequence alignments using Clustal Omega. Mol Syst Biol. 2011;7:539. 21988835 10.1038/msb.2011.75 PMC3261699

[45] Vijayabaskar MS, Vishveshwara S. Insights into the fold organization of TIM barrel from interaction energy based structure networks. PLOS Comput Biol. 2012;8(5): Article e1002505. 22615547 10.1371/journal.pcbi.1002505 PMC3355060

[46] Hossain MA, Higaki K, Saito S, Ohno K, Sakuraba H, Nanba E, Suzuki Y, Ozono K, Sakai N. Chaperone therapy for Krabbe disease: Potential for late-onset GALC mutations. J Hum Genet. 2015;60(9):539–545. 26108143 10.1038/jhg.2015.61

[47] McCarron EP, Oldham A, Herwadkar A, Jenkinson S, Campbell C, Neal K, Church HJ, Cooper JA, Stepien KM. Natural history and diagnostic findings in an adult man diagnosed with attenuated Krabbe disease. Am J Med Genet A. 2025;197(7): Article e64031. 40017455 10.1002/ajmg.a.64031

[48] Urizar E, McCarron EP, Gadepalli C, Bentley A, Woolfson P, Lin S, Iosifidis C, Browning AC, Bassett J, Senarathne UD, et al. Genetic insights and diagnostic challenges in highly attenuated lysosomal storage disorders. Genes. 2025;16(8):915. 40869962 10.3390/genes16080915 PMC12385809

[49] Casals N, Gómez-Puertas P, Pié J, Mir C, Roca R, Puisac B, Aledo R, Clotet J, Menao S, Serra D, et al. Structural (βα)_8_ TIM barrel model of 3-hydroxy-3-methylglutaryl-coenzyme A lyase. J Biol Chem. 2003;278(31):29016–29023. 12746442 10.1074/jbc.M304276200

[50] Wierenga RK. The TIM-barrel fold: A versatile framework for efficient enzymes. FEBS Lett. 2001;492(3):193–198. 11257493 10.1016/s0014-5793(01)02236-0

[51] Reardon D, Farber GK. The structure and evolution of α/β barrel proteins. FASEB J. 1995;9(7):497–503. 7737457 10.1096/fasebj.9.7.7737457

[52] Henrissat B, Callebaut I, Fabrega S, Lehn P, Mornon JP, Davies G. Conserved catalytic machinery and the prediction of a common fold for several families of glycosyl hydrolases. Proc Natl Acad Sci USA. 1995;92(15):7090–7094. 7624375 10.1073/pnas.92.15.7090 PMC41477

[53] Arya R, Lalloz MR, Bellingham AJ, Layton DM. Evidence for founder effect of the Glu104Asp substitution and identification of new mutations in triosephosphate isomerase deficiency. Hum Mutat. 1997;10(4):290–294. 9338582 10.1002/(SICI)1098-1004(1997)10:4<290::AID-HUMU4>3.0.CO;2-L

[54] Fermo E, Bianchi P, Vercellati C, Rees DC, Marcello AP, Barcellini W, Zanella A. Triose phosphate isomerase deficiency associated with two novel mutations in *TPI* gene. Eur J Haematol. 2010;85(2):170–173. 20374271 10.1111/j.1600-0609.2010.01451.x

[55] Hill CH, Cook GM, Spratley SJ, Fawke S, Graham SC, Deane JE. The mechanism of glycosphingolipid degradation revealed by a GALC-SapA complex structure. Nat Commun. 2018;9(1):151. 29323104 10.1038/s41467-017-02361-y PMC5764952

[56] Lee WC, Kang D, Causevic E, Herdt AR, Eckman EA, Eckman CB. Molecular characterization of mutations that cause globoid cell leukodystrophy and pharmacological rescue using small molecule chemical chaperones. J Neurosci. 2010;30(16):5489–5497. 20410102 10.1523/JNEUROSCI.6383-09.2010 PMC3278277

[57] Shteinberg M, Haq IJ, Polineni D, Davies JC. Cystic fibrosis. Lancet. 2021;397(10290):2195–2211. 34090606 10.1016/S0140-6736(20)32542-3

[58] Viuff A, Salamone S, McLoughlin J, Deane JE, Jensen HH. The bicyclic form of galacto-noeurostegine is a potent inhibitor of β-galactocerebrosidase. ACS Med Chem Lett. 2021;12(1):56–59. 33488964 10.1021/acsmedchemlett.0c00377 PMC7812600

