# Supplementary material for: Molecular Dynamics Simulations of 4 GALC Variants Causing Krabbe Disease
Source: Comput Struct Biotechnol J. 2026 May 20;35(1):0101. doi: 10.34133/csbj.0101 (PMC13187500; doi:10.34133/csbj.0101)
Supplement: Supplementary 1 — Figs. S1 to S14 [file csbj.0101.f1.pdf]

Molecular Dynamics Simulations of 4 GALC Variants Causing Krabbe Disease

Piet Ankermann[1], Silja I. Jenne[1], Jannes Talarek[1], Lukas Heger[2], Eileen Socher[1*]

[1]Institute of Functional and Clinical Anatomy, Friedrich-Alexander-Universität Erlangen-Nürnberg (FAU), Erlangen, Germany
[2]Department of Transfusion Medicine and Hemostaseology, Universitätsklinikum Erlangen, Friedrich-Alexander-Universität Erlangen-Nürnberg (FAU), Erlangen, Germany
*Correspondence: Eileen Socher, Institute of Functional and Clinical Anatomy, Friedrich-Alexander-Universität Erlangen-Nürnberg (FAU), Universitätsstraße 19, 91054 Erlangen, Germany, eileen.socher@fau.de

Supplementary Materials

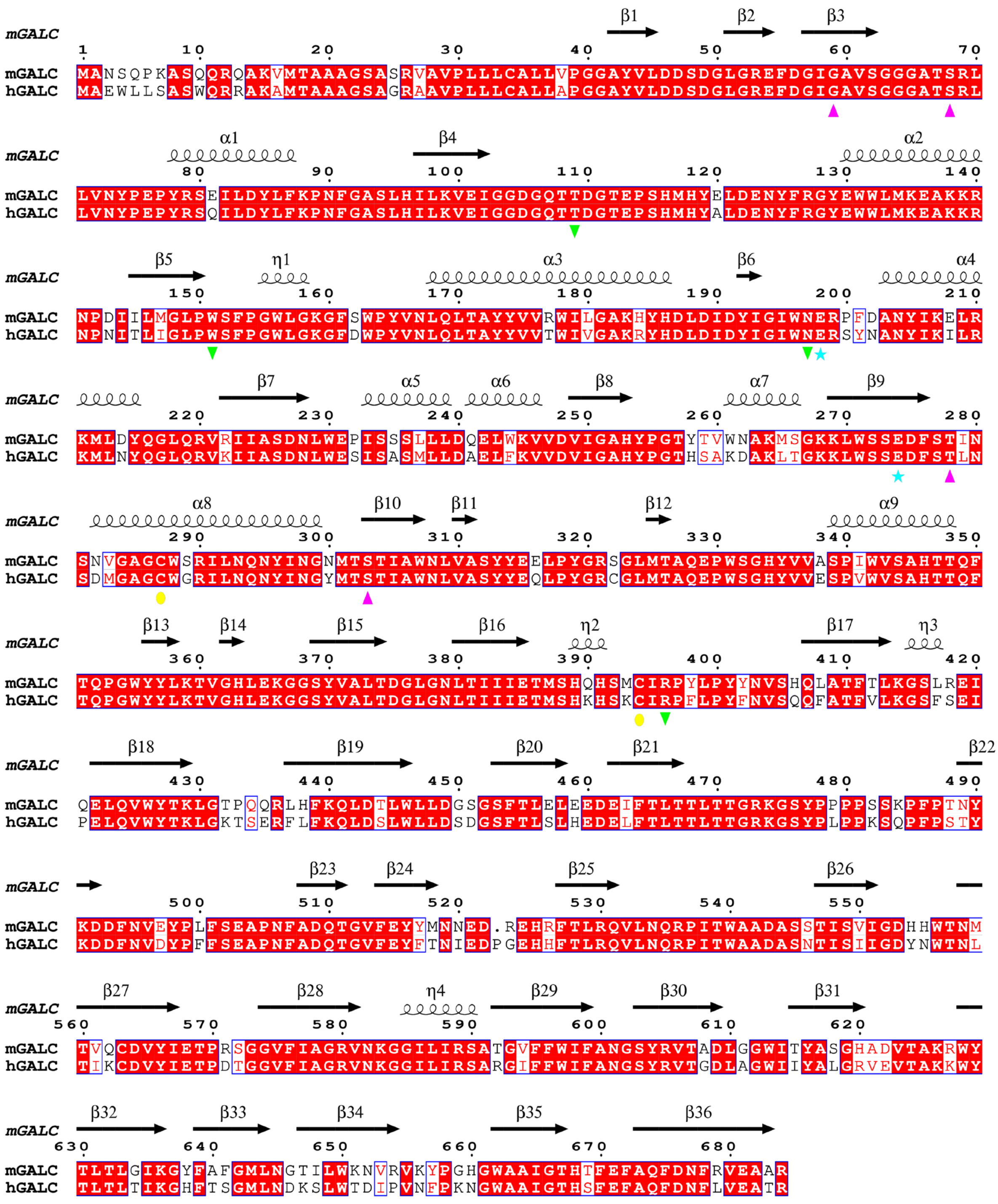

Fig. S1. Sequence alignment of human galactocerebrosidase (hGALC) and the orthologous murine galactocerebrosidase (mGALC). Identical amino acids are highlighted with white letters on a red background whereas similar amino acids are written with red letters. Secondary structure elements are indicated above the alignment (α for α-helices, η for 3_{10}-helices, and β for β-sheets) and numbered from the N terminus to the C terminus. Highlighted below the sequence alignment are the four residues corresponding to the four investigated mutations (purple triangles), the two cysteines forming the disulfide bond (yellow circles), the two catalytic residues Glu198 and Glue274 (blue stars) and the residues involved in substrate binding (Thr109, Trp151, Asn197 and Arg396; green triangles).

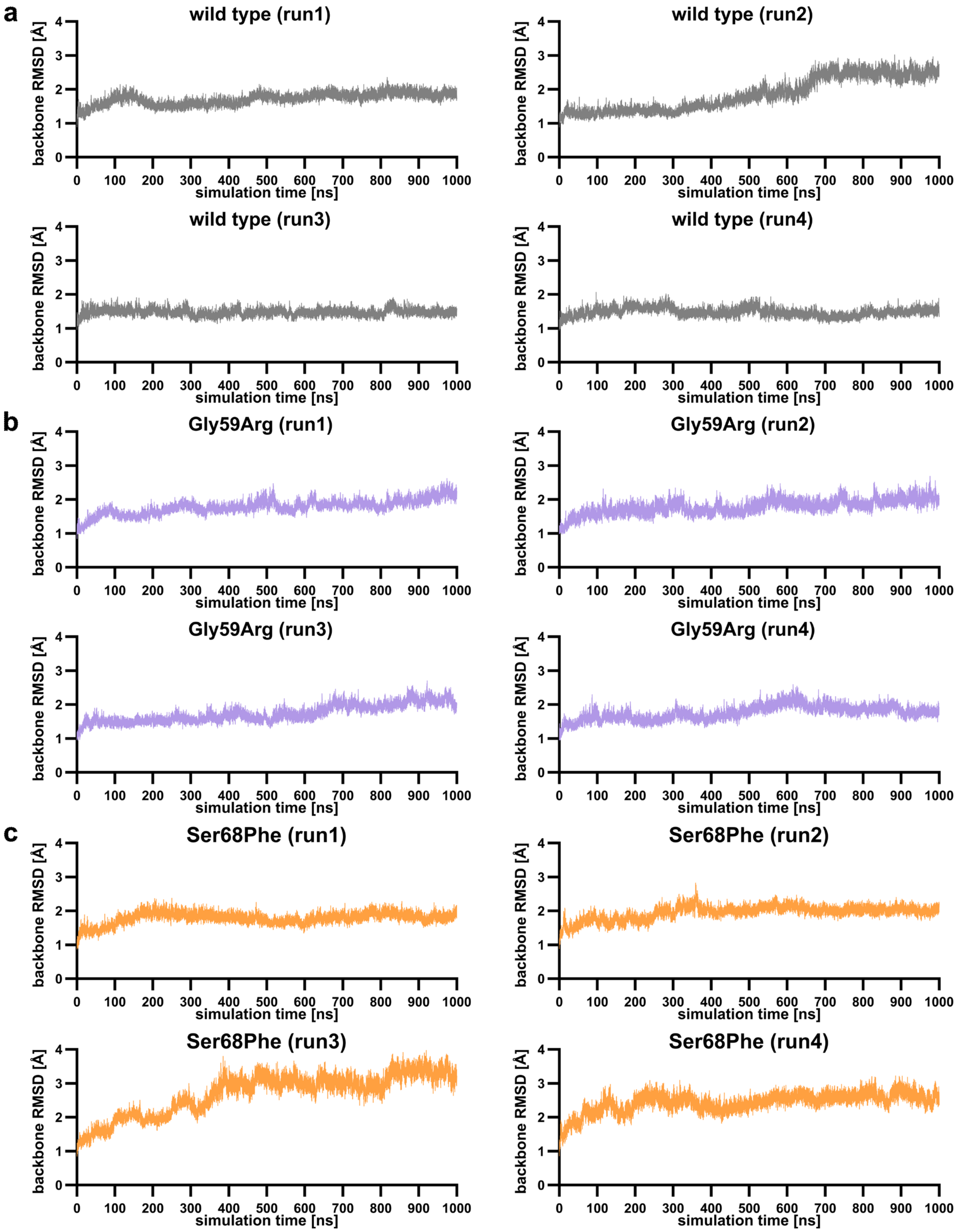

Fig. S2. Root-mean-square deviation (RMSD) of the protein backbone atoms in wild-type GALC and the GALC variants carrying the mutations Gly59Arg or Ser68Phe. Time-resolved RMSD plots for each simulation run describe the structural deviation between the protein structure at every time point during simulation and the starting structure: (**a**) wild-type GALC, (**b**) the Gly59Arg GALC variant, and (**c**) the Ser68Phe GALC variant.

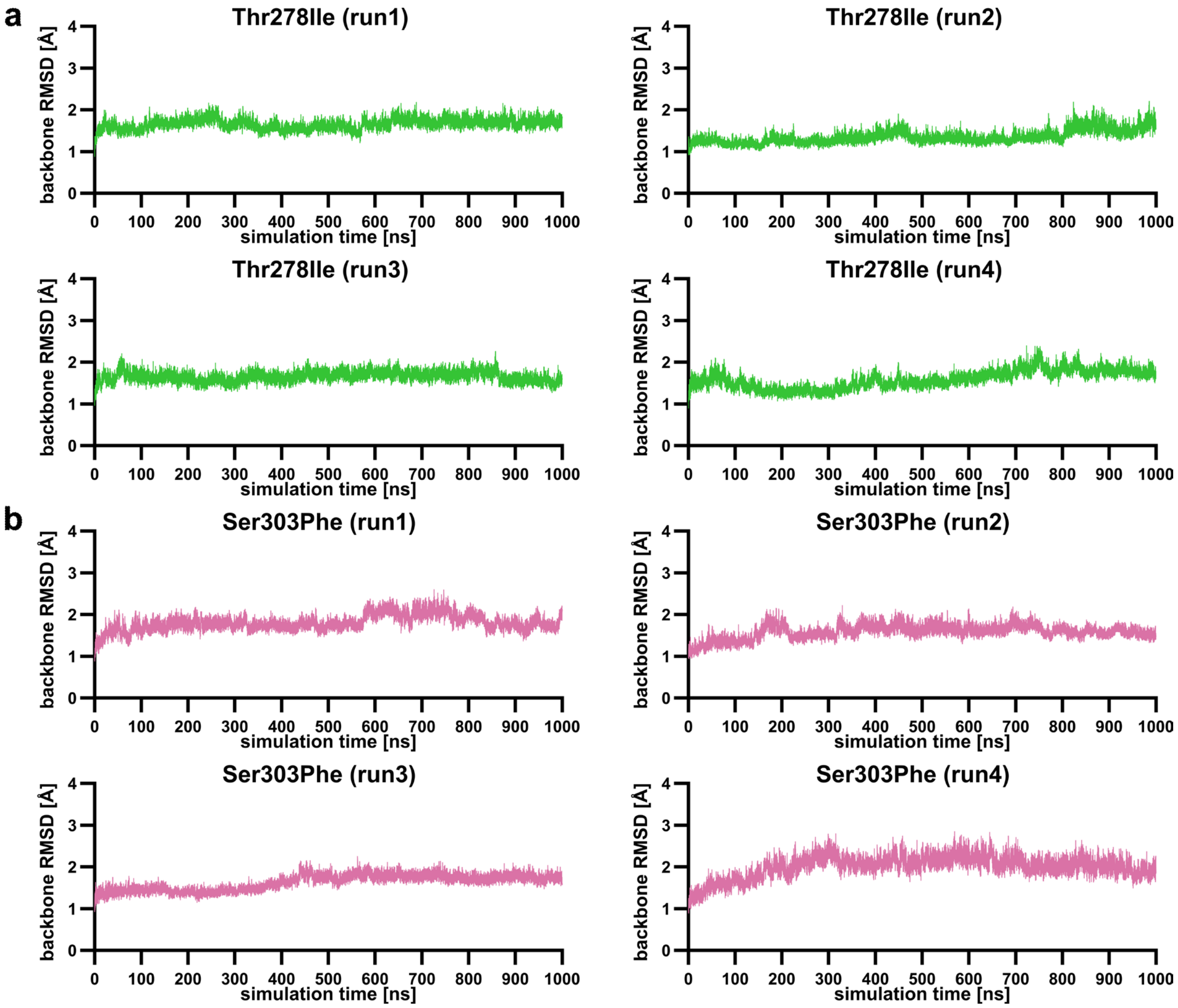

Fig. S3. Root-mean-square deviation (RMSD) of the protein backbone atoms in the GALC variants carrying the mutations Thr278Ile or Ser303Phe. Time-resolved RMSD plots for each simulation run describe the structural deviation between the protein structure at every time point during simulation and the starting structure: (**a**) the Thr278Ile GALC variant and (**b**) the Ser303Phe GALC variant.

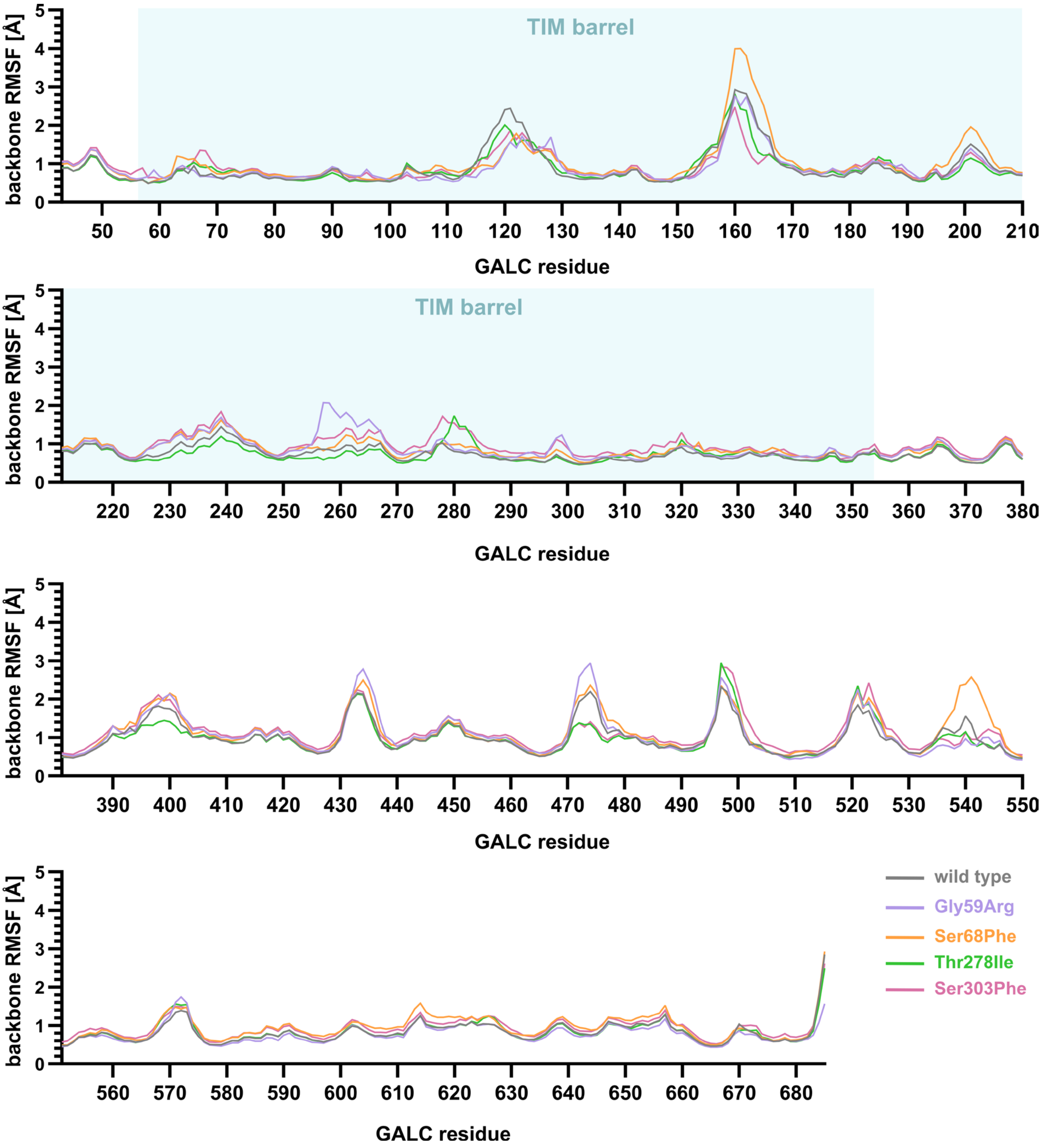

Fig. S4. Backbone fluctuations of human galactocerebrosidase (GALC) and the GALC variants Gly59Arg, Ser68Phe, Thr278Ile, Ser303Phe. The root-mean-square fluctuation (RMSF) values were calculated for the backbone atoms of each GALC residue. Mean of four independent simulation runs is shown.

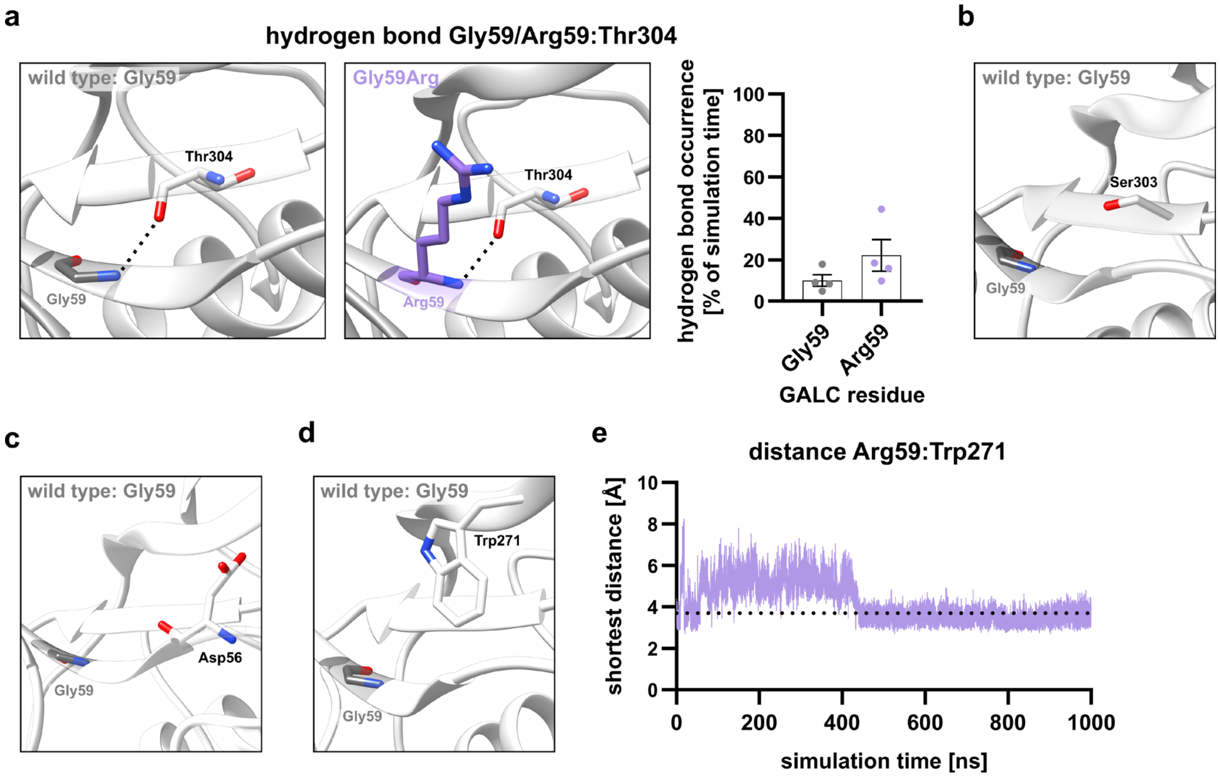

Fig. S5. Structural representations and local effects of different residue pairs interacting in the wild type and/or in the GALC variant Gly59Arg. (**a**) Structural representation of the hydrogen bond (indicated by a black dashed line) between the backbone of Gly59 or Arg59 and the backbone of Thr304. Hydrogen bond occurrence was measured for each simulation run of the wild type and the GALC variant with Gly59Arg and plotted as bar graphs. Mean values are shown as bar graphs ± SEM with circles representing individual simulation runs (n = 4). (**b-d**) In wild-type GALC, Gly59 has no functional groups in the side chain capable of forming hydrogen bonds, salt bridges, or cation–π interactions. Therefore, all possible side chain interactions formed by the Arg59 side chain in the GALC Gly59Arg variant are not possible in wild-type GALC. The corresponding figures, illustrating the respective situation in the GALC variant Gly59Arg, can be found in Fig. 3. (**e**) Time-resolved representative distance plot of the shortest distance for one of the simulation runs of the GALC variant Gly59Arg. The pooled results of all simulation runs are shown in Fig. 3g. The black dotted line indicates a distance of 3.7 Å, and if the measured distance is below this value, a cation-π interaction is likely present.

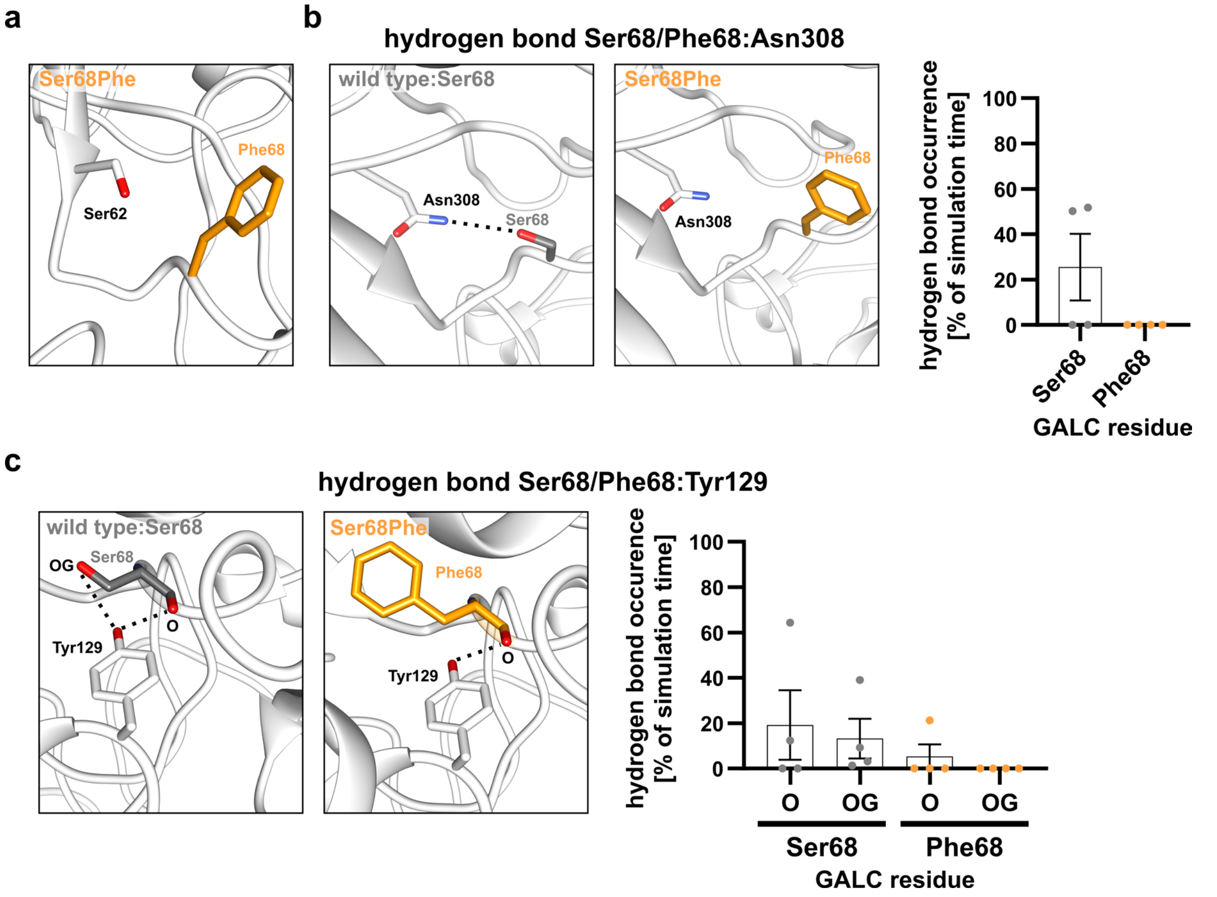

Fig. S6. Structural representations and local effects of different residue pairs interacting in the wild type and/or in the GALC variant Ser68Phe. (**a**) In the GALC variant Ser68Phe, the side chain-side chain hydrogen bond to Ser62 cannot be formed by a phenylalanine at position 68 as it cannot form any hydrogen bonds via its side chain. The corresponding figure, illustrating the respective situation in the wild type or the GALC variant Ser68Phe, can be found in Fig. 4. (**b**) Structural representation of the hydrogen bond (indicated by a black dashed line) between the amide group of Asn308 and the hydroxyl group of Ser68 in wild-type GALC. This interaction is not present in the Ser68Phe variant. Hydrogen bond occurrence was measured for each simulation run of the wild type and the GALC variant with Ser68Phe. (**c**) Structural representation of the hydrogen bonds (indicated by black dashed lines) between the hydroxyl group of Tyr129 and the backbone of Ser68 or Phe68 and the side chain hydroxyl group of Ser68 in wild-type GALC. Hydrogen bond occurrence was measured as in (b). (**b,c**) Mean values are shown as bar graphs ± SEM with circles representing individual simulation runs (n = 4).

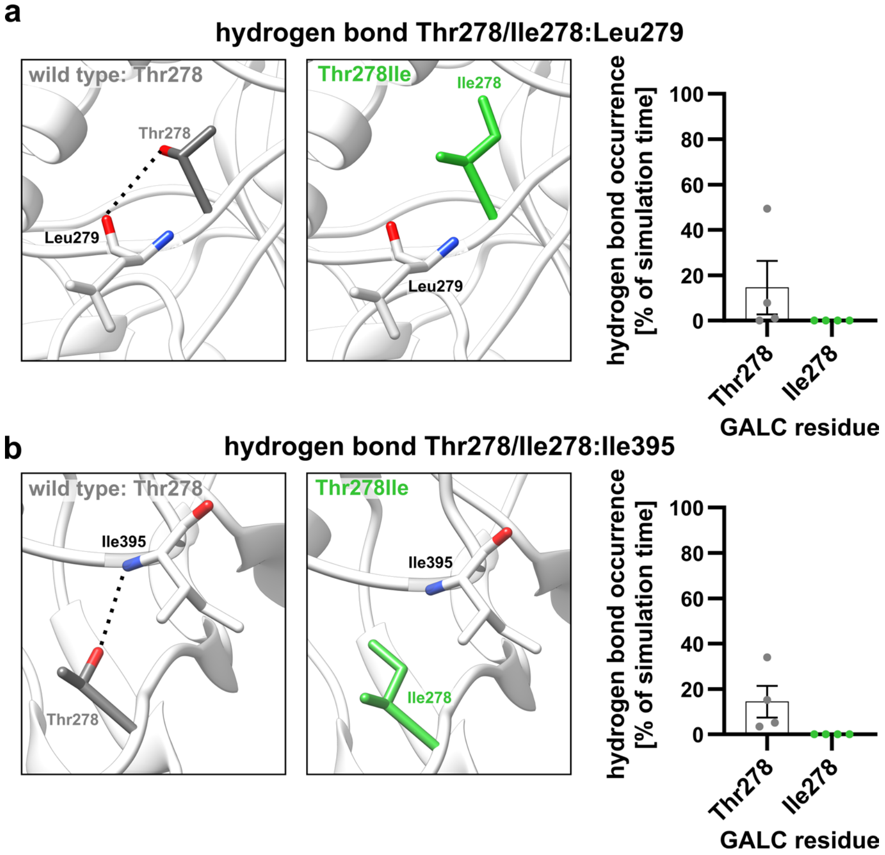

Fig. S7. Structural representations and local effects of different residue pairs interacting in the wild-type GALC and not in the GALC variant Thr278Ile. (**a**) Structural representation of the hydrogen bond (indicated by a black dashed line) between the side chain of Thr278 and the backbone of Leu279. This interaction is only present in wild-type GALC and cannot be formed by an isoleucine side chain at position 278 because the nonpolar isoleucine side chain is unable to form hydrogen bonds. Hydrogen bond occurrence was measured for each simulation run of the wild type and the GALC variant Thr278Ile. (**b**) Structural representation of the hydrogen bond (indicated by a black dashed line) between the side chain of Thr278 and the backbone of Ile395. This interaction is only present in wild-type GALC for the same reason as in (a). Hydrogen bond occurrence was measured as in (a). (**a**,**b**) Mean values are shown as bar graphs ± SEM with circles representing individual simulation runs (n = 4).

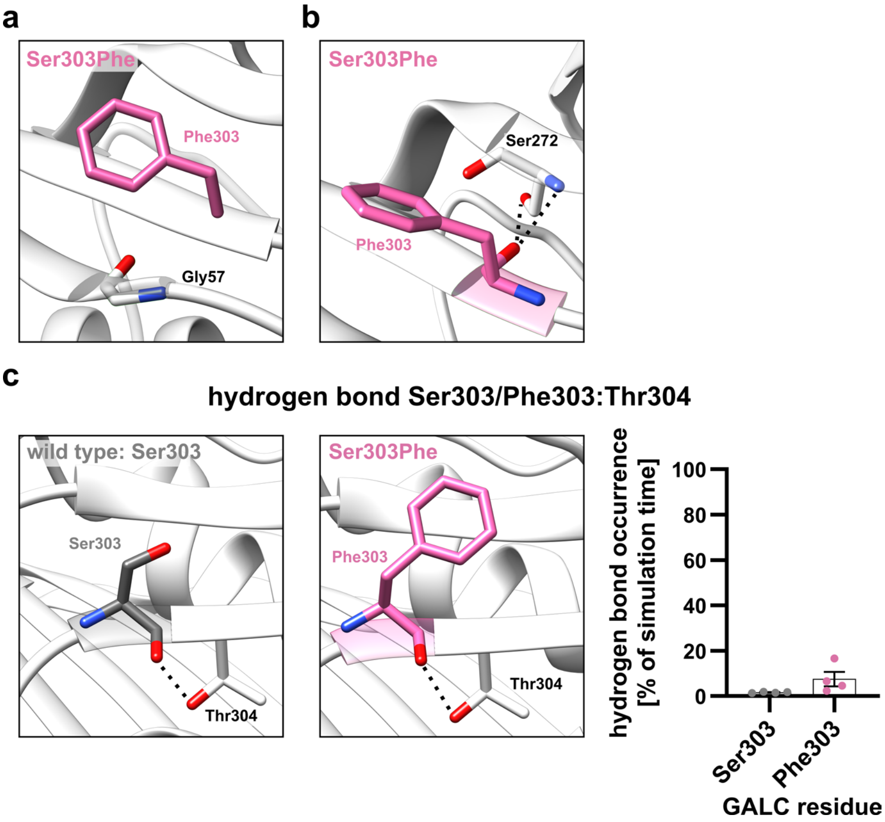

Fig. S8. Structural representations of different residue pairs interacting in the wild type and/or in the GALC variant Ser303Phe. (**a**) In the GALC variant Phe303Ser, the hydrophobic phenylalanine side chain has no hydrogen bond to Gly57. (**b**) However, the phenylalanine backbone can form hydrogen bonds with Ser272. The corresponding figures for (a) and (b), illustrating the respective situation in wild-type GALC, can be found in Fig. 6. (**c**) Structural representation of the hydrogen bond (indicated by a black dashed line) between the backbone carbonyl group of Ser303/Phe303 with the side chain hydroxyl group of Thr304. Hydrogen bond occurrence was measured for each simulation run of the wild type and GALC variant Ser303Phe. Mean values are shown as bar graphs ± SEM with circles representing individual simulation runs (n = 4).

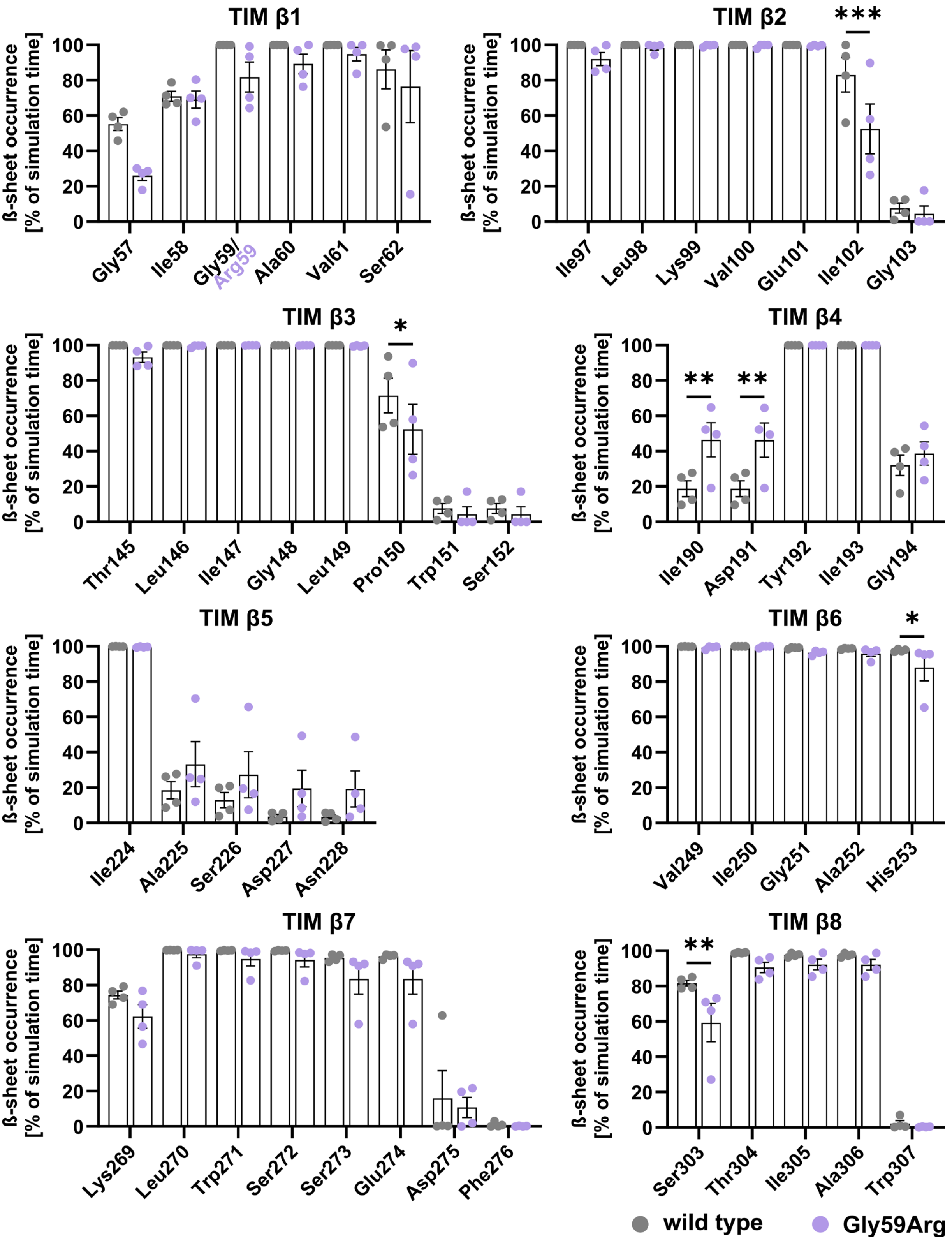

Fig. S9. β-Sheet occurrence of individual residues within the eight β-strands (TIM β1-TIM β8) constituting the inner β-barrel of the TIM barrel in the GALC Gly59Arg variant. For each residue, two columns are shown: one representing the wild type (gray) and one representing the Gly59Arg mutation (purple). Each of the four data points represents the mean β-sheet occurrence for one simulation run. Mean values ± SEM are displayed as bar graphs (n = 4). Statistical analysis was performed in GraphPad Prism (v10) using 2way ANOVA with Šídák's multiple comparisons test (*p < 0.05, **p < 0.01, ***p < 0.001).

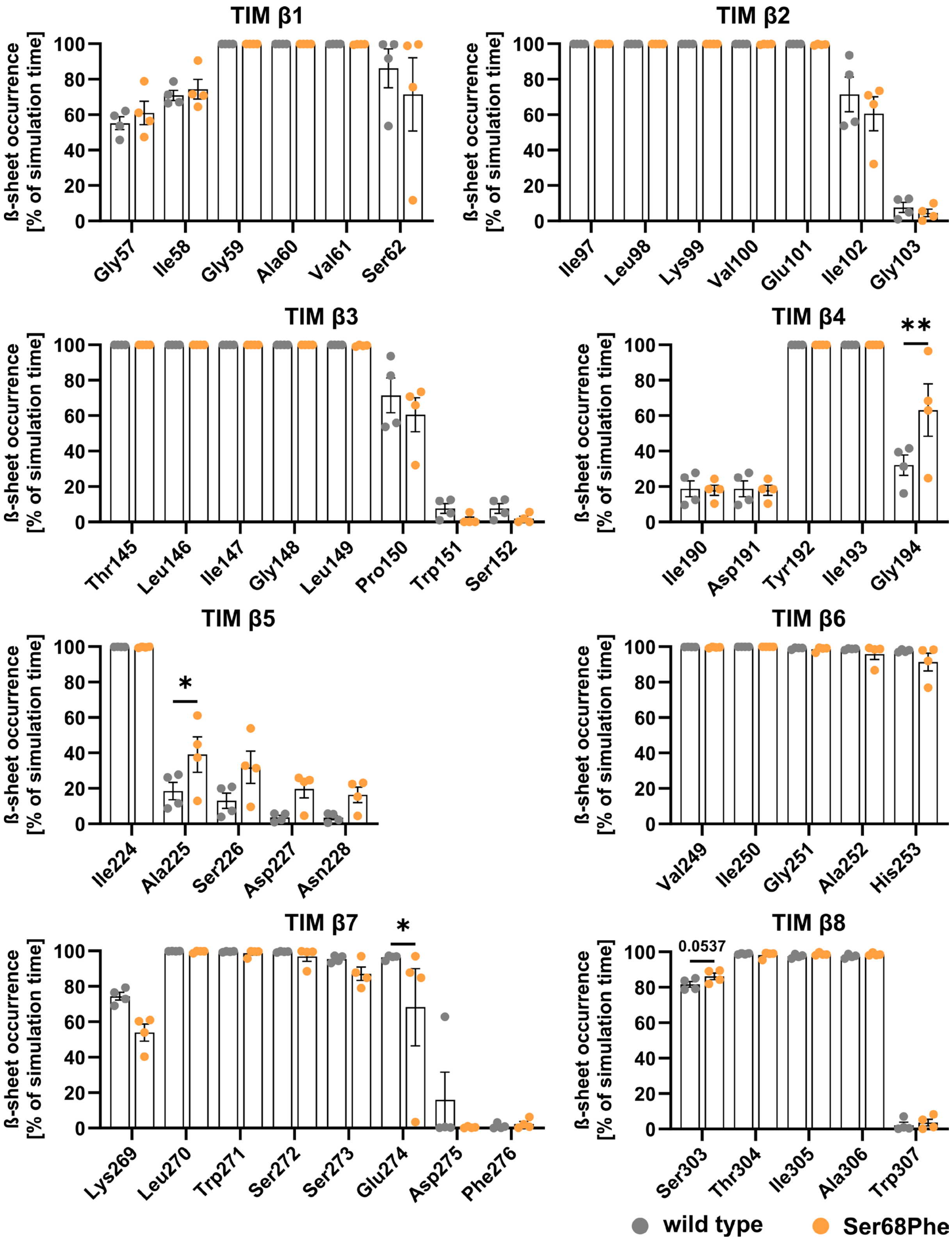

Fig. S10. β-Sheet occurrence of individual residues within the eight β-strands (TIM β1-TIM β8) constituting the inner β-barrel of the TIM barrel in the GALC Ser68Phe variant. For each residue, two columns are shown: one representing the wild type (gray) and one representing the Ser68Phe mutation (orange). Each of the four data points represents the mean β-sheet occurrence for one simulation run. Mean values ± SEM are displayed as bar graphs (n = 4). Statistical analysis was performed in GraphPad Prism (v10) using 2way ANOVA with Šídák's multiple comparisons test (*p < 0.05, **p < 0.01).

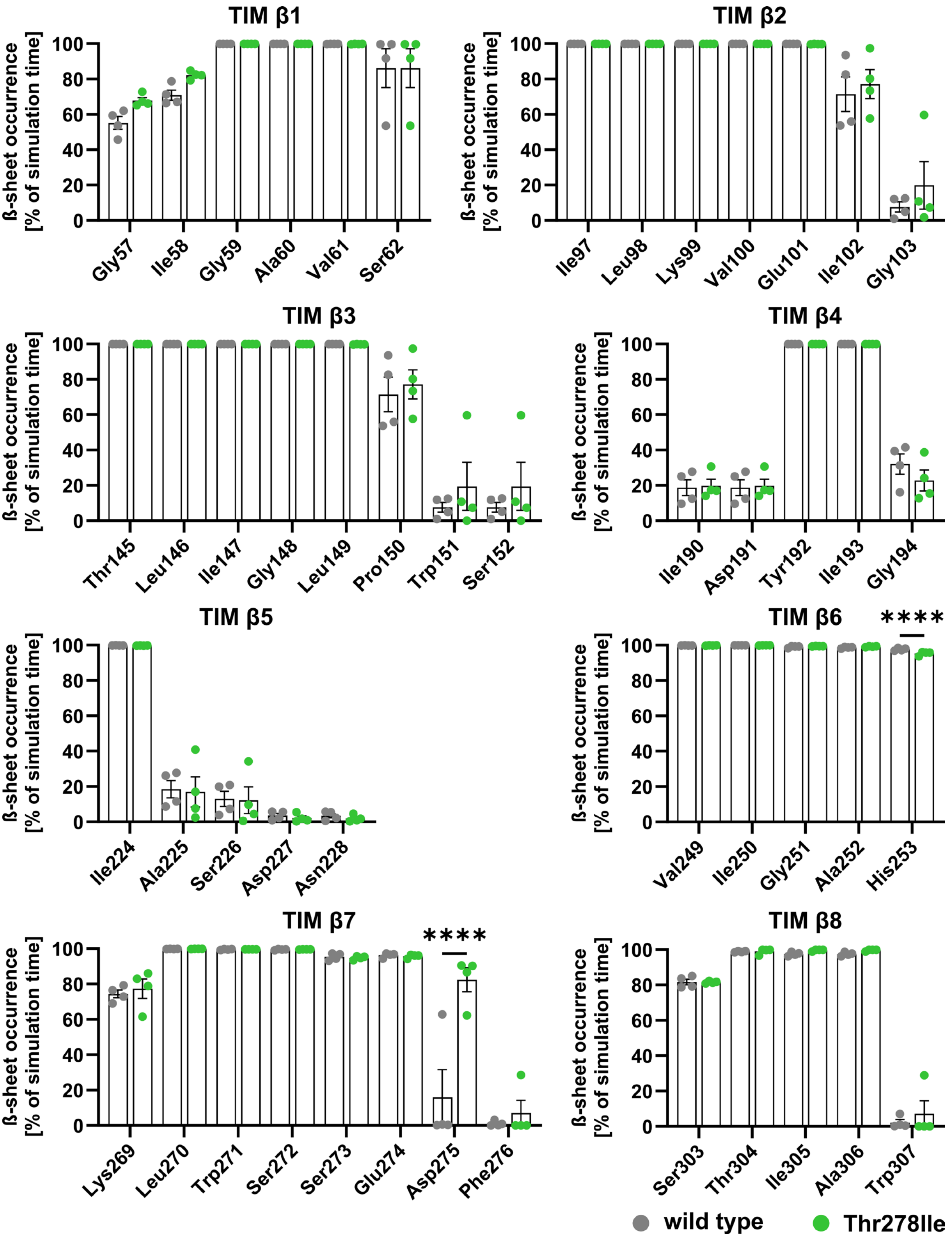

Fig. S11. β-Sheet occurrence of individual residues within the eight β-strands (TIM β1-TIM β8) constituting the inner β-barrel of the TIM barrel in the GALC Thr278Ile variant. For each residue, two columns are shown: one representing the wild type (gray) and one representing the Thr278Ile mutation (green). Each of the four data points represents the mean β-sheet occurrence for one simulation run. Mean values ± SEM are displayed as bar graphs (n = 4). Statistical analysis was performed in GraphPad Prism (v10) using 2way ANOVA with Šídák's multiple comparisons test (****$p < 0.0001$).

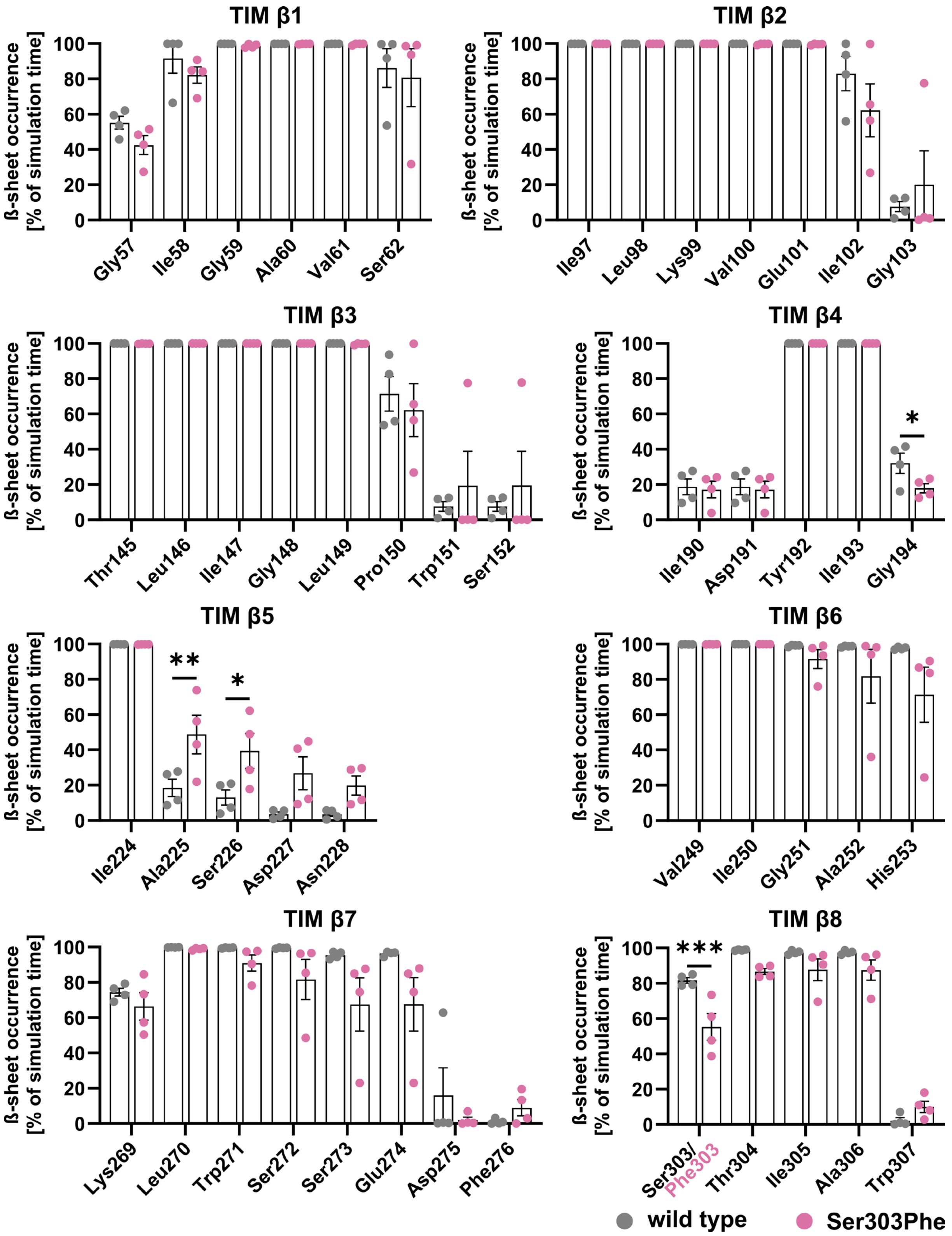

Fig. S12. β-Sheet occurrence of individual residues within the eight β-strands (TIM β1-TIM β8) constituting the inner β-barrel of the TIM barrel in the GALC Ser303Phe variant. For each residue, two columns are shown: one representing the wild type (gray) and one representing the Ser303Phe mutation (pink). Each of the four data points represents the mean β-sheet occurrence for one simulation run. Mean values ± SEM are displayed as bar graphs (n = 4). Statistical analysis was performed in GraphPad Prism (v10) using 2way ANOVA with Šídák's multiple comparisons test (*p < 0.05, **p < 0.01, ***p < 0.001).

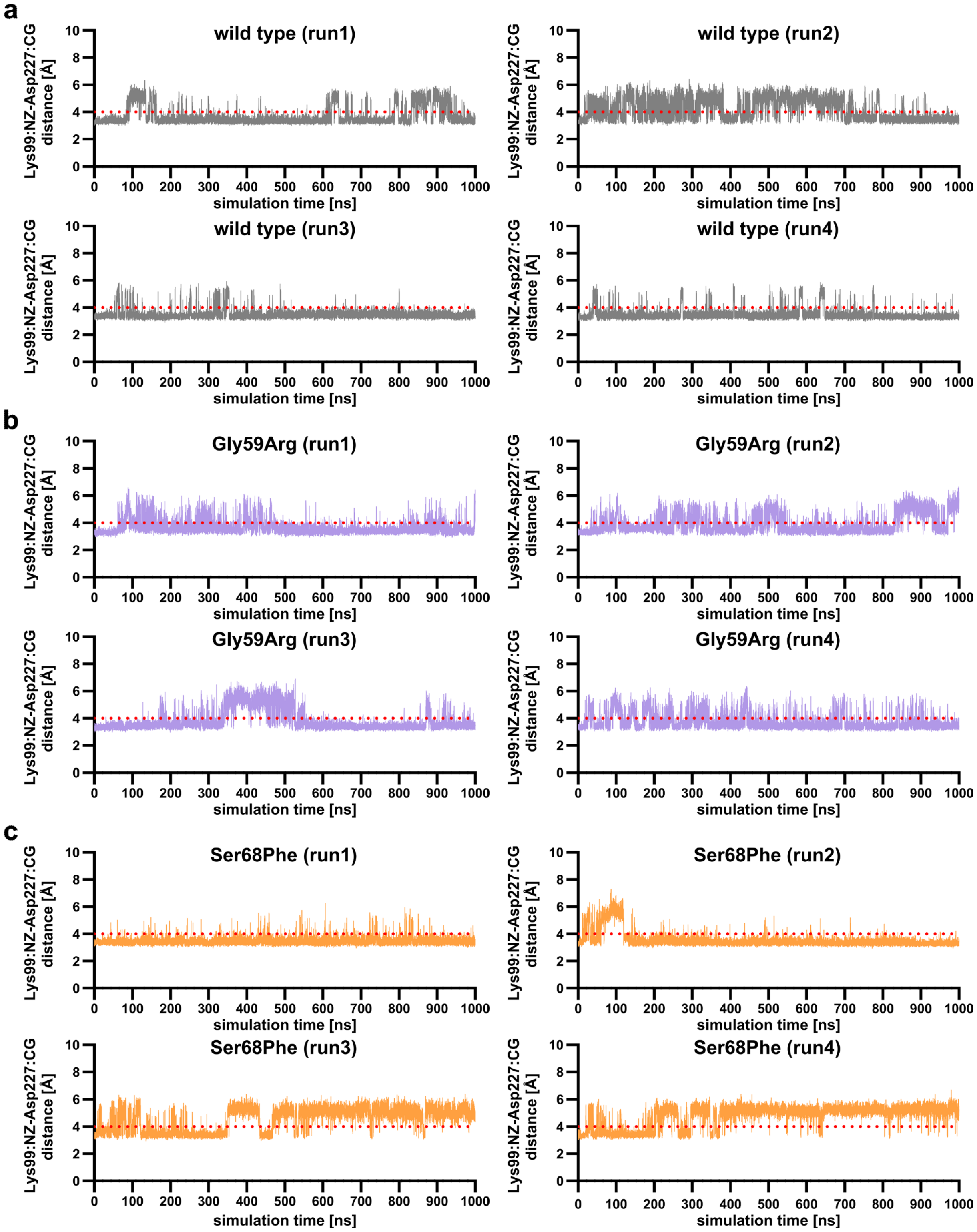

Fig. S13. Salt bridge between the side chains of Lys99 and Asp227 in wild-type GALC and the GALC variants with the mutation Gly59Arg or Ser68Phe. Time-resolved distance plots for every simulation run in order to describe the distance between the Lys99:NZ atom and the Asp227:CG atom (**a**) in wild-type GALC, (**b**) the Gly59Arg GALC variant and (**c**) the Ser68Phe GALC variant. The red dotted lines indicate a distance of 4 Å, and if the measured distance is below this value, a salt bridge is likely present.

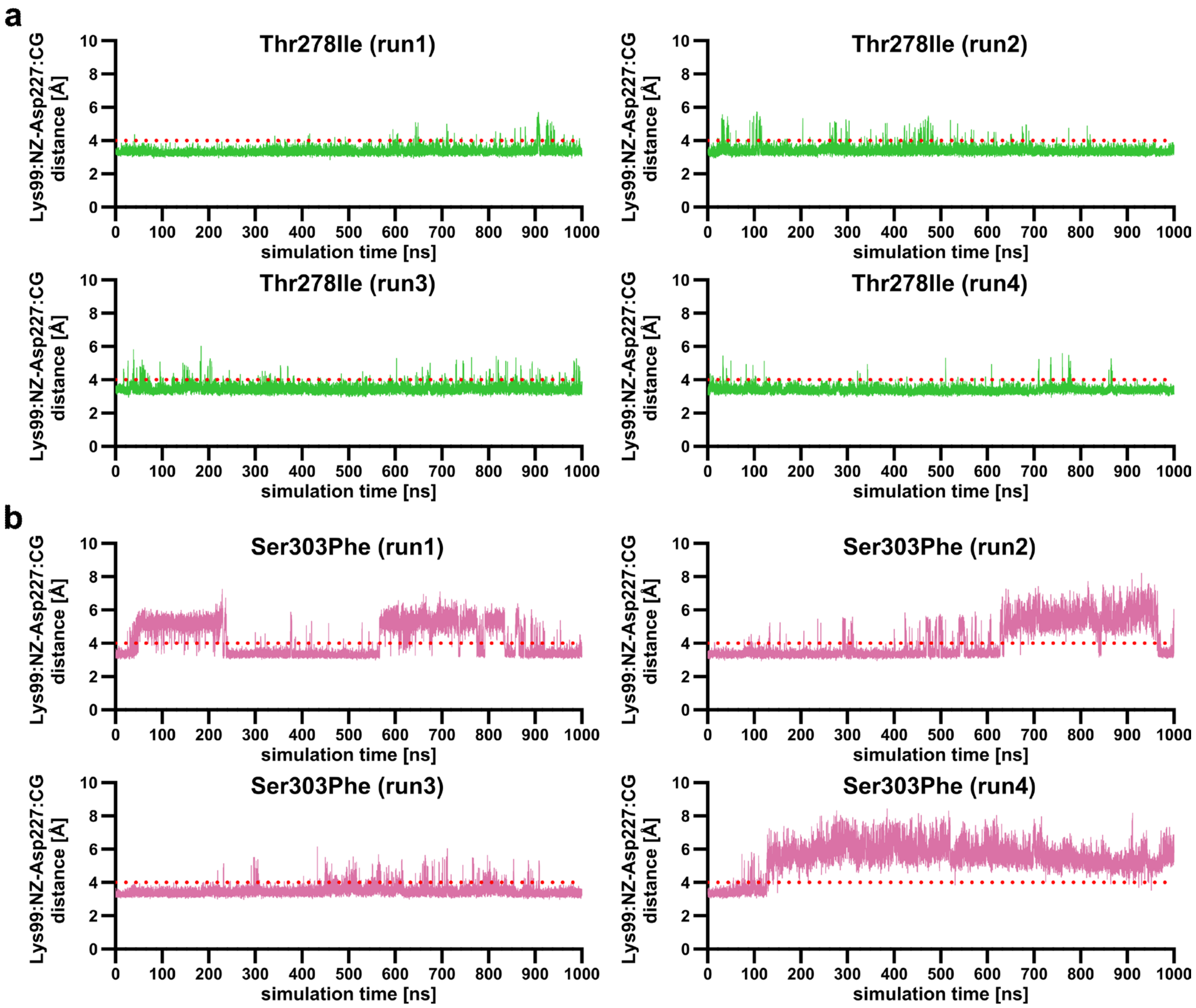

Fig. S14. Salt bridge between the side chains of Lys99 and Asp227 in the GALC variants with the mutation Thr278Ile or Ser303Phe. Time-resolved distance plots for every simulation run to describe the distance between the Lys99:NZ atom and the Asp227:CG atom (**a**) in the Thr278Ile GALC variant and (**b**) the Ser303Phe GALC variant. The red dotted lines indicate a distance of 4 Å, and if the measured distance is below this value, a salt bridge is likely present.
